# Biocatalytic reversible control of the stiffness of DNA-modified responsive hydrogels: applications in shape-memory, self-healing and autonomous controlled release of insulin†

**DOI:** 10.1039/d0sc01319f

**Published:** 2020-04-14

**Authors:** Chen Wang, Amit Fischer, Avner Ehrlich, Yaakov Nahmias, Itamar Willner

**Affiliations:** Institute of Chemistry, The Minerva Center for Bio-hybrid Complex Systems, The Hebrew University of Jerusalem Jerusalem 91904 Israel Itamar.willner@mail.huji.ac.il; Grass Center for Bioengineering, Benin School of Computer Science and Engineering, The Hebrew University of Jerusalem 91904 Israel

## Abstract

The enzymes glucose oxidase (GOx), acetylcholine esterase (AchE) and urease that drive biocatalytic transformations to alter pH, are integrated into pH-responsive DNA-based hydrogels. A two-enzyme-loaded hydrogel composed of GOx/urease or AchE/urease and a three-enzyme-loaded hydrogel composed of GOx/AchE/urease are presented. The biocatalytic transformations within the hydrogels lead to the dictated reconfiguration of nucleic acid bridges and the switchable control over the stiffness of the respective hydrogels. The switchable stiffness features are used to develop biocatalytically guided shape-memory and self-healing matrices. In addition, loading of GOx/insulin in a pH-responsive DNA-based hydrogel yields a glucose-triggered matrix for the controlled release of insulin, acting as an artificial pancreas. The release of insulin is controlled by the concentrations of glucose, hence, the biocatalytic insulin-loaded hydrogel provides an interesting sense-and-treat carrier for controlling diabetes.

## Abstract 2

Biocatalytic control over the stiffness of pH-responsive hydrogels is applied to develop shape-memory, self-healing and controlled release matrices.

## Graphical abstract

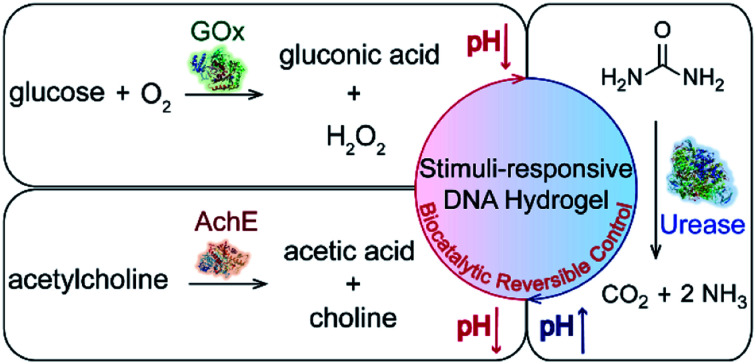

## Introduction

Stimulus-responsive hydrogels undergoing gel-to-liquid, gel-to-solid or gel-transitions between variable stiffness states attract continuous interest due to their diverse potential applications.^1–6^ Different external triggers were applied to stimulate phase-transitions of hydrogels or to control their stiffness properties. These include pH,^7^ heat,^8,9^ light,^10–13^ chemical agents^14,15^ and magnetic fields.^16^ Different applications of stimulus-responsive hydrogels were suggested, including their use as controlled drug-delivery carriers,^17–19^ sensing,^20^ tissue repair,^21,22^ bone-healing materials,^23,24^ and actuating and robotic devices.^25–29^ Within the broad class of stimulus-responsive hydrogels, stimulus-responsive nucleic acid-bridged hydrogels, *i.e.* hydrogels that consist of polyacrylamide or carboxymethyl cellulose crosslinked by nucleic acids, find growing interest.^30,31^ Besides the crosslinking of polymer chains by means of duplex nucleic acid bridges,^32^ hydrogels cooperatively stabilized by duplex nucleic acids and stimulus-responsive nucleic acid crosslinking units exhibit signal-triggered stiffness functions.^33,34^ For example, cytosine-rich strands undergo reversible pH-triggered reconfiguration between random strands and i-motif structures.^35^ Guanosine-rich nucleic acids undergo, in the presence of K^+^-ions/crown ethers, reversible transitions between G-quadruplex and random strand configurations,^36^ nucleic acid strands functionalized with photoisomerizable azobenzene units demonstrate light-induced formation and dissociation of duplex nucleic acids upon photoisomerization of the azobenzene units between *trans* and *cis* states,^27,37,38^ and triplex T–A·T or C–G·C^+^ nucleic acids undergo pH-stimulated reconfiguration of the nanostructures.^39,40^ Indeed, hydrogels cooperatively stabilized by duplex nucleic acid bridges and signal-triggered, reconfigurable cross-linkers were reported, demonstrating control over the stiffness of the hydrogels by means of pH,^39,41,42^ K^+^-ions/crown ethers^43,44^ or light.^45–47^ Also, aptamer-modified hydrogels were reported as functional matrices for the release of protein drugs.^48^ Recently, light-induced control over the stiffness of hydrogels through incorporation of plasmonic Au nanoparticles/Au nanorods into duplex nucleic acid-bridged hydrogels was reported.^49^ These Au nanoparticle-functionalized hydrogels enable thermoplasmonic heating, resulting in the thermal separation of the nucleic acid bridges and control over the stiffness of the hydrogel matrices. In addition, stimulus-responsive hydrogels were suggested to enable various applications, including the development of shape-memory,^39,41,50,51^ self-healing matrices,^15,46,47^ assembly of triggered and switchable drug release materials^52^ and carriers,^53^ the design of hydrogels exhibiting triggered mechanical bending properties,^54^ the control over ion-transport through nanopores,^55^ and the development of switchable electrocatalytic hydrogel materials.^43,44^

**Fig. 1:**
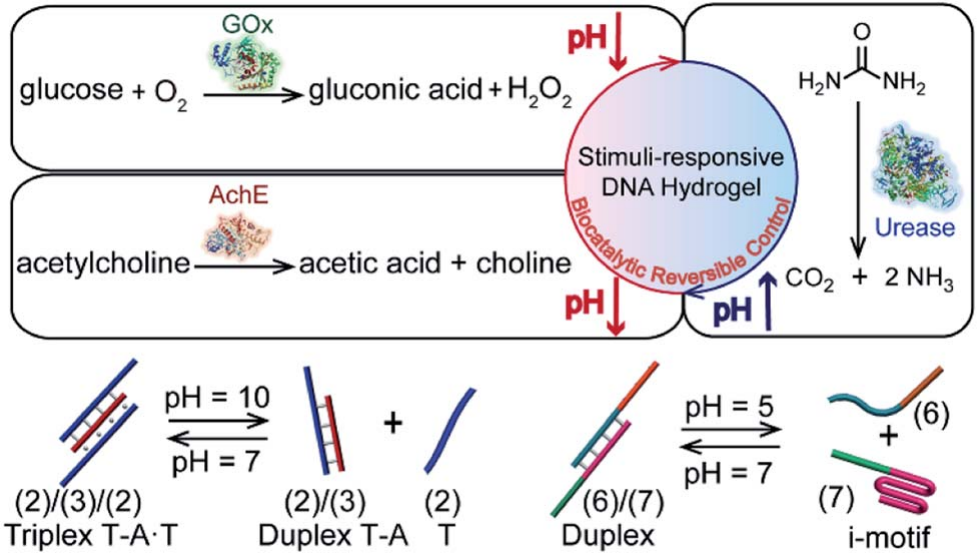
Schematic reversible biocatalytic control of pH in stimulus-responsive DNA-based hydrogels using three different biocatalysts (GOx, AchE, and urease) and two different motifs (T–A·T triplex and i-motif structures) as reconfigurable cross-linkers.

Here we report the incorporation of enzymes into DNA-based hydrogels to yield biocatalytic control over the stiffness of the matrices, especially, we demonstrate the integration of glucose oxidase (GOx), acetylcholine esterase (AchE) and urease in polyacrylamide hydrogels cooperatively stabilized by two pH-responsive crosslinking motifs (Fig. 1). The biocatalytic control of pH in hydrogels, in response to the respective substrates, leads to the reversible switchable control over the stiffness of the matrices. The applications of the biocatalyst-functionalized hydrogels as shape-memory, self-healing and controlled drug release matrices are demonstrated. Specifically, we introduce GOx and insulin into a pH-responsive DNA-based hydrogel. Glucose-guided pH changes in the GOx-loaded hydrogel lead to the release of insulin. The hydrogel mimics the functions of the pancreas, revealing a concentration-dependent insulin release in response to glucose; an important attribute for glucose management in diabetes. It should be noted that numerous studies addressed the incorporation of enzymes in hydrogels as functional matrices for operating biotechnological applications^56^ or sensing,^57^ and enzyme-loaded hydrogels were used as functional matrices for the biocatalysed, triggered degradation of the matrices and release of loads.^58^ Nonetheless, the enzyme-loaded DNA-based hydrogels, where the biocatalysts are retained and caged in the hydrogels within the process of the biocatalytic control over the stiffness of the hydrogels (and the accompanying shape-memory/self-healing events), are, to the best of our knowledge, unprecedented. Such enzyme-triggered, non-degradable hydrogels are anticipated to introduce new stimulus-responsive materials. Furthermore, the reversible enzyme-driven release of loads from the hydrogels may have important biomedical applications reflected by switchable drug release and the elimination of immunogenic responses. The unique switchable reconfiguration of nucleic acids^59^ suggests that the coupling of enzymes with stimulus-responsive hydrogel materials leads to functional materials that feature shape-memory, self-healing and controlled drug-release properties.

## Results and discussion

**Fig. 2:**
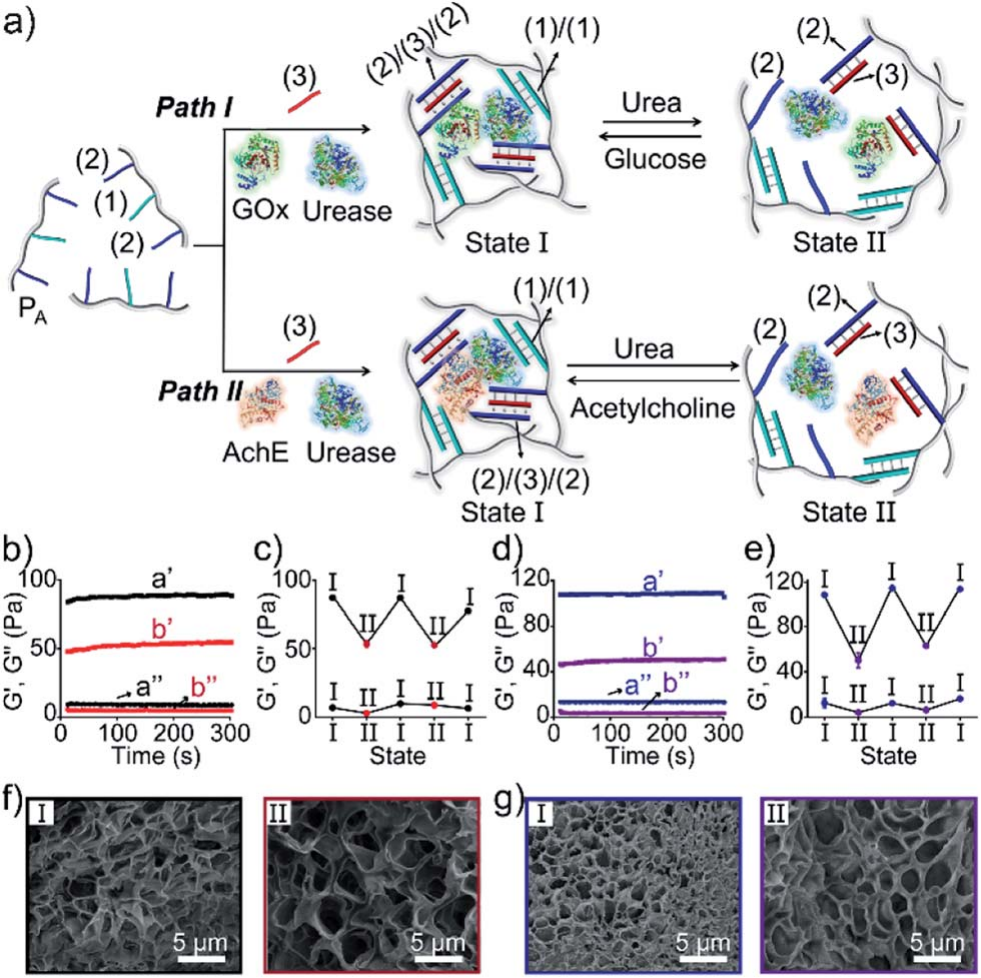
(a) Preparation and reversible control over the stiffness of the GOx/urease (path I) or AchE/urease-loaded (path II) DNA-based hydrogels using the switchable, biocatalytic, pH-stimulated reconfiguration of T–A·T triplex cross-linkers. (b) Rheometric features of the GOx/urease-loaded hydrogel in the presence of glucose (a′/a′′) and urea (b′/b′′). (c) Switchable stiffness properties of the hydrogel (path I) in the presence of glucose (state I) and urea (state II). (d) Rheometric features of the AchE/urease-loaded hydrogel in the presence of acetylcholine (a′/a′′) and urea (b′/b′′). (e) Switchable stiffness properties of the hydrogel (path II) in the presence of acetylcholine (state I) and urea (state II). (f) SEM images of the hydrogel (path I) in the presence of glucose (panel I) and urea (panel II). (g) SEM images of the hydrogel (path II) in the presence of acetylcholine (panel I) and urea (panel II).

The preparation and properties of the first stimulus-responsive biocatalytic hydrogel system are presented in Fig. 2. The polyacrylamide scaffold P_A_ was prepared by the copolymerization of acrylamide and the acrydite nucleic acid monomers (**1**) and (**2**) (see the ESI†). The loading of the nucleic acids ((**1**) + (**2**)) on the polymer chains was evaluated by UV spectroscopy to be 1 : 58 (DNA : acrylamide), Fig. S1.† The tether (**1**) includes a self-complementary sequence, while tether (**2**) is designed to generate a T–A·T triplex in the presence of the strand (**3**). The polymer P_A_, in the presence of the strand (**3**), glucose oxidase (GOx) and urease, yields a GOx/urease-loaded hydrogel cooperatively crosslinked by (**1**)/(**1**) duplexes and (**2**)/(**3**)/(**2**) T–A·T triplexes (path I). An additional, related, biocatalytic hydrogel included the loading of acetylcholine esterase (AchE) and urease in the (**1**)/(**1**) and (**2**)/(**3**)/(**2**) cooperatively crosslinked hydrogel (path II). The loading of the enzymes GOx, urease and AchE in the hydrogels corresponds to 23, 28 and 27 units, respectively (details in the ESI and Fig. S2†). For the synthetic details for the preparation of different nucleic acid-modified hydrogels, the evaluation of nucleic acid loads on different polymers and the detailed sequences of the respective DNA used in the present study and details on the evaluation of the contents of the different biocatalysts in the respective hydrogel matrices, see the Experimental section, ESI.† The mechanisms to switch the stiffness of the hydrogels by the loaded biocatalysts are presented in Fig. 1 and 2a. Treatment of the GOx/urease- or AchE/urease-loaded hydrogels with urea led to the urease-stimulated hydrolysis of urea to ammonia, causing an increase of pH within the hydrogels (see pH changes in Table S2†). This resulted in the separation of the T–A·T triplex cross-linkers and the hydrogels bridged only by the (**1**)/(**1**) units. The lower degree of crosslinking yielded hydrogels with lower stiffness. Treatment of the lower-stiffness hydrogel with glucose (path I) resulted in the aerobic oxidation of glucose to gluconic acid and H_2_O_2_. The biocatalytically formed gluconic acid acidified the hydrogel, regenerating the stiffer hydrogel crosslinked by the (**1**)/(**1**) duplex and the (**2**)/(**3**)/(**2**) triplex bridges. Similarly, subjecting the lower-stiffness hydrogel to acetylcholine (path II) led to the formation of choline and acetic acid; the process acidified the hydrogel and restored the hydrogel of higher stiffness cooperatively stabilized by the (**1**)/(**1**) and (**2**)/(**3**)/(**2**) bridging motifs. The enzyme-driven and switchable stiffness properties of the hydrogels were confirmed by rheometric measurements. Fig. 2b reveals that the GOx/urease-loaded hydrogel crosslinked by (**1**)/(**1**) and T–A·T cross-linkers exhibits *G*′ and *G*′′ values corresponding to 90 Pa and 9 Pa (curve a′ and a′′). The treatment of the hydrogel with urea led to the urease-catalyzed hydrolysis of urea and to the pH-stimulated dissociation of the T–A·T triplexes, resulting in a hydrogel with lower stiffness, *G*′ = 55 Pa and *G*′′ = 5 Pa (curve b′ and b′′). The reversible treatment of the hydrogel with urea and glucose allowed switchable control over the stiffness of the hydrogel (Fig. 2c). Similar results were observed for the hydrogel loaded with AchE and urease. The initial hydrogel crosslinked cooperatively by (**1**)/(**1**) and (**2**)/(**3**)/(**2**) revealed a higher stiffness, *G*′ = 110 Pa and *G*′′ = 12 Pa, curve a′ and a′′ as shown in Fig. 2d. Treatment of the hydrogel with urea led to a lower-stiffness hydrogel, *G*′ = 50 Pa and *G*′′ = 4 Pa (curve b′ and b′′). Similarly, the cyclic treatment of the hydrogel with urea and acetylcholine switched the stiffness of the hydrogel between lower and higher values (Fig. 2e). Scanning electron microscopy (SEM) images of the hydrogels in different states further support the stiffness properties of the hydrogels. In Fig. 2f and g, panels I show the SEM images of the stiff hydrogels loaded with GOx/urease and AchE/urease (state I), respectively, and dense small-pore matrices are observed, consistent with the higher crosslinking degree of the hydrogels. Panels II show in Fig. 2f and g, the SEM images of the two hydrogels after treatment with urea (state II). Large pores of lower density are observed, consistent with the lower crosslinking degree of the hydrogels. It should be noted that the pH changes stimulated by the enzyme-loaded hydrogels are controlled by the concentrations of enzymes and respective substrates in the matrices. The loading degrees specified above represent optimized loading degrees that stimulate the desired pH changes within a predefined time-interval. For example, the discussion and Tables S1–S4 in the ESI† describe the pH changes of the GOx/urease and AchE/urease matrices, in the presence of variable contents of the biocatalysts.

**Fig. 3:**
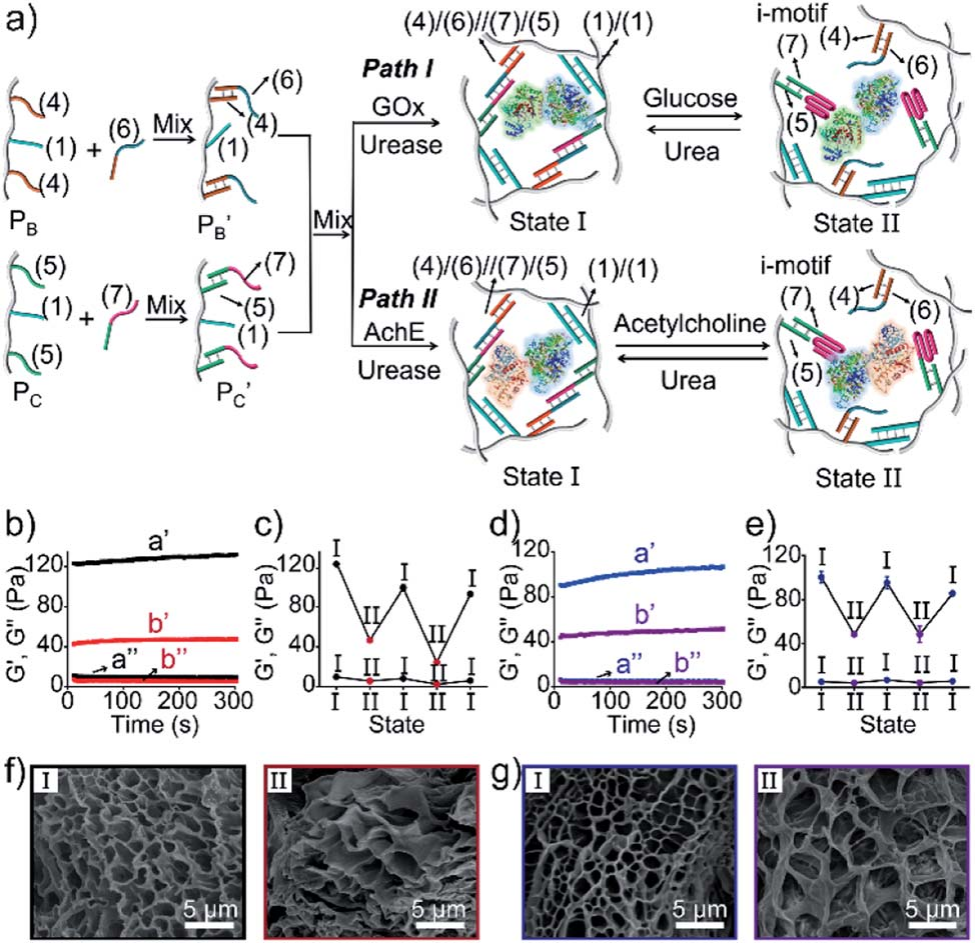
(a) Preparation and switchable stiffness control of the GOx/urease (path I) or AchE/urease-loaded (path II) hydrogels using the reversible pH-induced formation and dissociation of i-motif units. (b) Rheometric features of the GOx/urease-loaded hydrogel in the presence of urea (a′/a′′) and glucose (b′/b′′). (c) Switchable stiffness properties of the hydrogel (path I) in the presence of urea (state I) and glucose (state II). (d) Rheometric features of the AchE/urease-loaded hydrogel in the presence of urea (a′/a′′) and acetylcholine (b′/b′′). (e) Switchable stiffness properties of the hydrogel (path II) in the presence of urea (state I) and acetylcholine (state II). (f) SEM images of the hydrogel (path I) in the presence of urea (panel I) and glucose (panel II). (g) SEM images of the hydrogel (path II) in the presence of urea (panel I) and acetylcholine (panel II).

Besides the biocatalytically stimulated pH-controlled stiffness of the hydrogels by means of T–A·T triplex responsive bridges, the design of biocatalytic matrices that control their stiffness by pH-responsive i-motif structures was also demonstrated, as shown in Fig. 3. Two polyacrylamide chains 
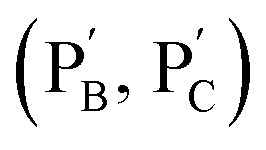
 functionalized with nucleic acids were prepared. In the first step, polymer P_B_ modified with the self-complementary strand (**1**) and the strand (**4**), and polymer P_C_ functionalized with the nucleic acid strands (**1**) and (**5**) were prepared (see the loading of DNA in Fig. S3†). In the next step, the strand (**6**) was hybridized with the strand (**4**) to yield polymer 
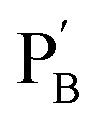
 consisting of duplexes extended by single-strand toehold domains. Similarly, the strand (**5**) associated with P_C_ was hybridized with strand (**7**) to yield a polymer 
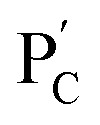
 consisting of duplexes, where the hybridized tether (**7**) includes a single-strand domain. The single-strand domains associated with the tethers (**6**) and (**7**) are complementary to each other. Mixing the two polymers 
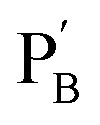
 and 
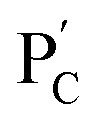
, in the presence of the enzymes, yielded the hydrogels cooperatively crosslinked by the (**1**)/(**1**) bridges and by the duplexes generated between the single-strand domains of (**6**) and (**7**) associated with (**4**)/(**6**) and (**5**)/(**7**) duplexes linked to 
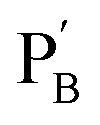
 and 
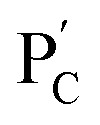
, respectively. Strand (**7**) in 
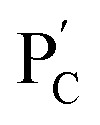
 participating in the bridging of the chains is, however, engineered to include a cytosine-rich sequence capable to form the i-motif structure at acidic pH. This provides the principle for biocatalytically guided control over the stiffness of the hydrogels, as outlined in Fig. 3a. Subjecting the GOx/urease-loaded hydrogel to glucose led to the aerobic-GOx-catalyzed oxidation of glucose to gluconic acid and H_2_O_2_ (path I), while subjecting the AchE/urease matrix to acetylcholine resulted in the hydrolysis of acetylcholine to choline and acetic acid (path II). The product, gluconic acid or acetic acid, acidifies the hydrogels to state II, due to the partial reconfiguration of sequence (**7**) into the i-motif structure and the dissociation of the (**6**)//(**7**) duplexes. Treatment of the lower-stiffness hydrogels with urea resulted in the urease-catalyzed hydrolysis of urea to ammonia that neutralizes the hydrogels. This resulted in the dissociation of the i-motif structures and the regeneration of the high-stiffness hydrogels cooperatively crosslinked by the (**1**)/(**1**) duplexes and the (**4**)/(**6**)//(**7**)/(**5**) bridges. Thus, by the cyclic biocatalytic activation of the GOx/urease or AchE/urease-loaded hydrogels cooperatively crosslinked by the (**1**)/(**1**) and (**4**)/(**6**)//(**7**)/(**5**) bridges and the partial pH-stimulated dissociation of one of the bridges *via* the formation of i-motif units, the hydrogels were reversibly switched between higher- and lower-stiffness states. The quantitative stiffness properties of the hydrogels were evaluated by rheometry, as shown in Fig. 3b–e. The GOx/urease-loaded hydrogel cooperatively stabilized by (**1**)/(**1**) and (**4**)/(**6**)//(**7**)/(**5**) cross-linkers revealed *G*′ = 124 Pa and *G*′′ = 10 Pa (curve a′ and a′′), and the glucose-induced separation of (**4**)/(**6**)//(**7**)/(**5**) by the formation of i-motif units yielded a softer hydrogel, *G*′ = 46 Pa, *G*′′ = 6 Pa, curve b′ and b′′ as shown in Fig. 3b. By the cyclic treatment of the hydrogel with glucose and urea, the stiffness of the hydrogel was reversibly switched, as shown in Fig. 3c. Similarly, the AchE/urease-loaded hydrogel, cooperatively bridged by the two crosslinking motifs, revealed a higher stiffness *G*′ = 100 Pa and *G*′′ = 5 Pa (curve a′ and a′′), as compared to the i-motif dissociated hydrogel that showed lower stiffness, *G*′ = 49 Pa, *G*′′ = 4 Pa, curve b′ and b′′ in Fig. 3d. Additionally, the AchE/urease-loaded hydrogel treated reversibly with acetylcholine and urea shows switchable stiffness transitions, as can be seen in Fig. 3e. The biocatalytic control over the stiffness features of the hydrogels was further supported by SEM images, (Fig. 3f and g). The hydrogels in state I showed dense small-pore structures (panels I), consistent with the high crosslinking degree of the hydrogel matrices; the respective glucose or acetylcholine treated hydrogels showed lower density of larger-pores (panels II), consistent with the lower degree of crosslinking of the matrices.

**Fig. 4:**
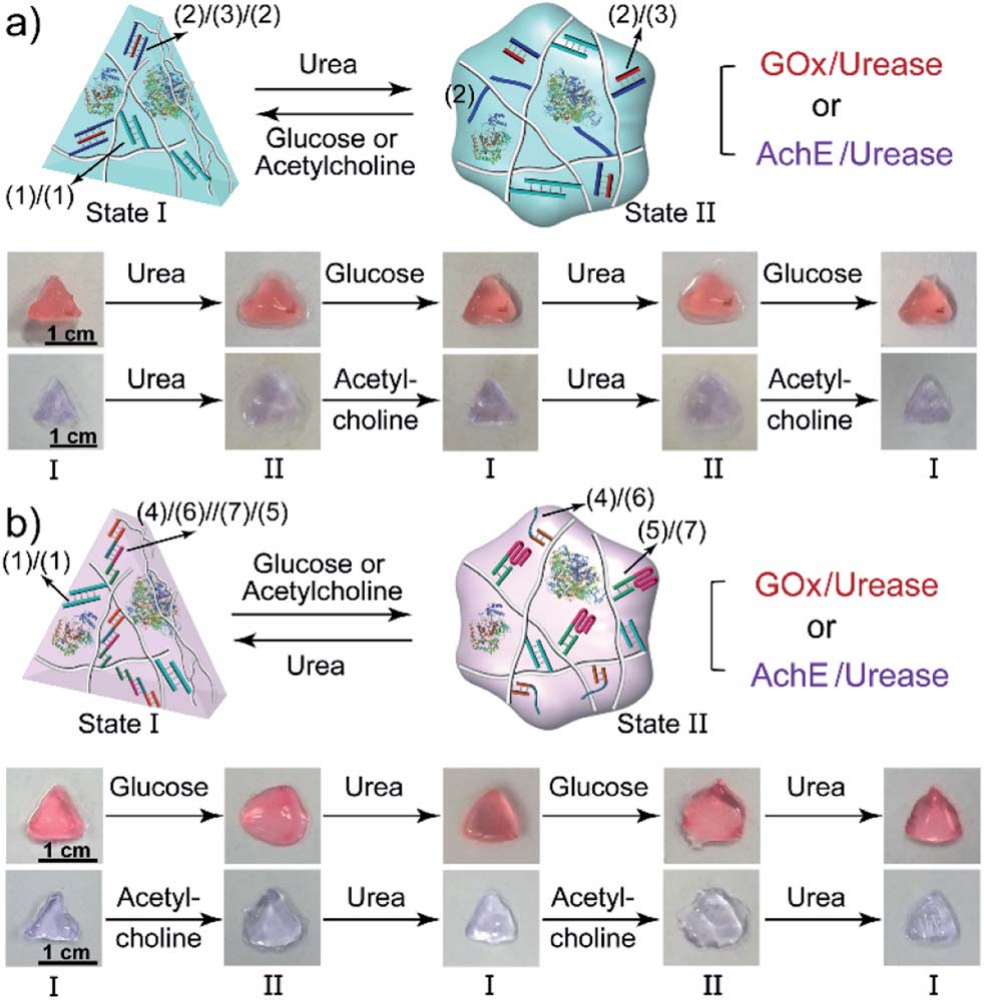
(a) Biocatalytically guided reversible shape-memory properties of the GOx/urease or AchE/urease-loaded hydrogels (*cf.*Fig. 2a) (the shapes are preserved in the presence of glucose or acetylcholine). (b) Biocatalytically guided reversible shape-memory properties of the GOx/urease- or AchE/urease-loaded hydrogels (*cf.*Fig. 3a) (the shapes are preserved in the presence of urea).

The biocatalyzed control over the stiffness of the different hydrogels was then used to develop hydrogel matrices exhibiting shape-memory properties. That is, subjecting a shaped, higher-stiffness hydrogel, crosslinked by the duplexes (**1**)/(**1**) and the enzyme-responsive bridges, to the respective substrate transforms the hydrogel into a lower-stiffness, quasi-liquid state that is stabilized only by the (**1**)/(**1**) duplex bridges. The residual duplex bridges provide, however, a memory reflected by the dictated entanglement of the polymer chains. Upon the counter biocatalyzed regeneration of the cooperatively bridged hydrogels, the duplex “memory” units provide instructive information to regenerate the shaped, higher-stiffness hydrogels. This is exemplified in Fig. 4a with the application of GOx/urease- or AchE/urease-loaded hydrogels, cooperatively crosslinked by the (**1**)/(**1**) duplexes and (**2**)/(**3**)/(**2**) triplexes, as biocatalytic shape memory matrices. The stiff biocatalyst-loaded hydrogels were prepared in molds and were extruded as a triangle. Subjecting the hydrogels to urea separated the triplex bridges, resulting in lower-stiffness, shapeless matrices crosslinked only by the duplexes (**1**)/(**1**). Treatment of the quasi-liquid, shapeless matrices with glucose or acetylcholine resulted in the neutralization of the hydrogels, and the memory-guided reshaping of triangle-shaped hydrogels stabilized by the two cooperative crosslinking units. In analogy, Fig. 4b demonstrates the reversible shape-memory of the GOx/urease- or AchE/urease-loaded hydrogels cooperatively stabilized by the (**1**)/(**1**) and (**4**)/(**6**)//(**7**)/(**5**) cross-linkers. The reversible transitions of the triangle-shaped and the shapeless, quasi-liquid hydrogels were realized upon the treatment of the hydrogels with glucose/urea or acetylcholine/urea.

**Fig. 5:**
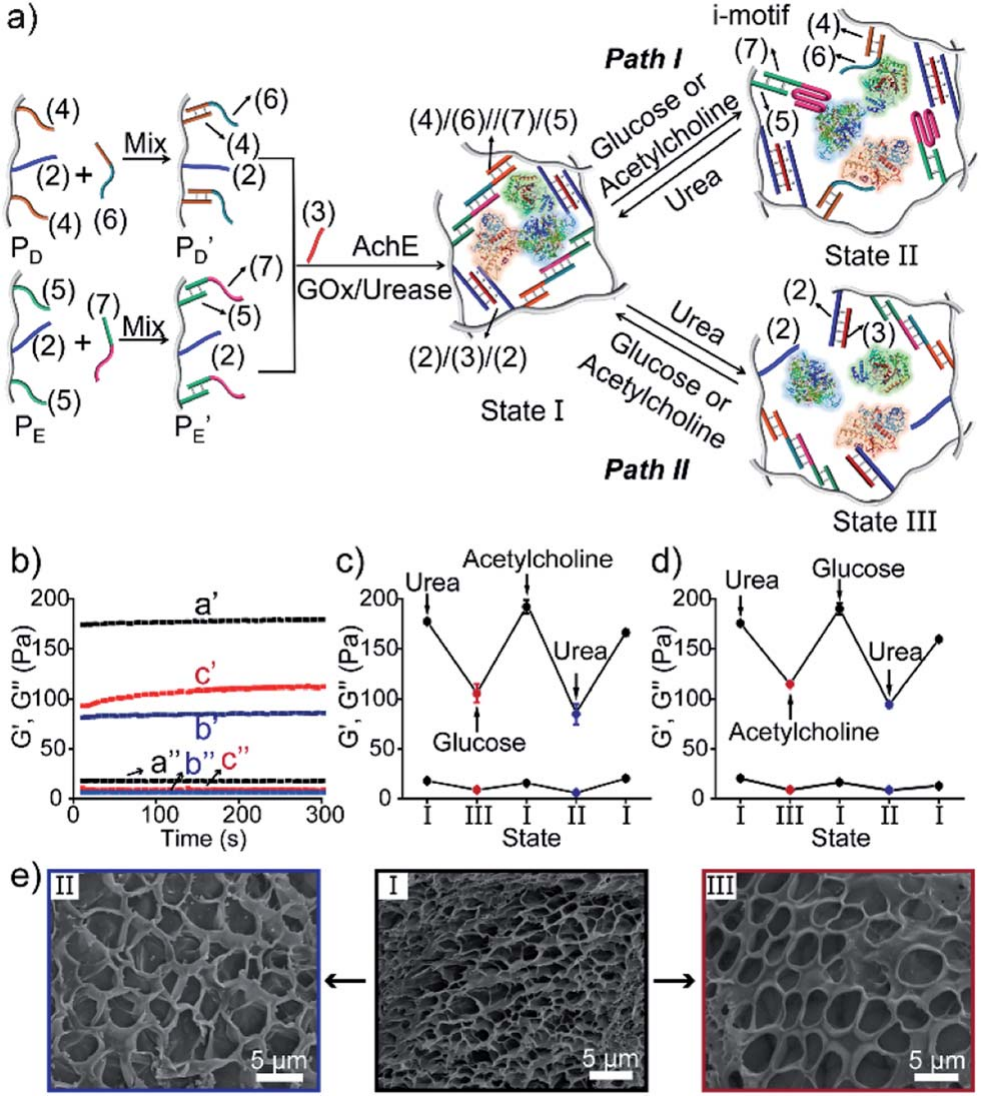
(a) Assembly of a three-enzyme-loaded DNA-based hydrogel (enzymes: GOx, AchE, and urease) undergoing biocatalyst-dictated reversible stiffness changes, in the presence of urea, glucose or acetylcholine, through the reconfiguration of T–A·T/duplex or duplex/i-motif structures. (b) Rheometric features of the three-enzyme-loaded hydrogel: a′/a′′ the stiff hydrogel in state I; b′/b′′ the stiff hydrogel treated with glucose or acetylcholine (state II); c′/c′′ the stiff hydrogel treated with urea (state III). (c) Cyclic transitions of the three-enzyme-loaded hydrogel in the presence of urea, glucose, acetylcholine and urea. (d) Cyclic transitions of the three-enzyme-loaded hydrogel in the presence of urea, acetylcholine, glucose, urea. (e) SEM images corresponding to: panel I – hydrogel in state I; panel II – hydrogel in state II; panel III – hydrogel in state III.

**Fig. 6:**
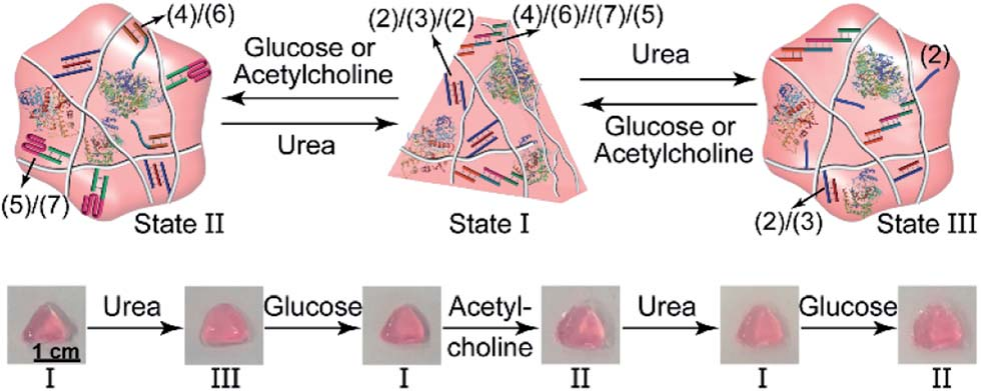
Cyclic shape-memory features of the three-enzyme-loaded hydrogel crosslinked by T–A·T and supramolecular duplex units guided by urea, glucose or acetylcholine.

In addition, all three enzymes GOx, AchE and urease were integrated into one hydrogel cooperatively crosslinked by the (**2**)/(**3**)/(**2**) T–A·T triplexes and the (**4**)/(**6**)//(**7**)/(**5**) bridges (Fig. 5a) (see Fig. S4† for the DNA loading on P_D_ and P_E_). This hydrogel reveals switchable stiffness properties in the presence of three substrates, *i.e.*, glucose, acetylcholine and urea. In the presence of glucose or acetylcholine (path I), the acidification of the higher-stiffness hydrogel (state I) leads to the separation of the (**6**)//(**7**) duplex units into the i-motif structures and the formation of the lower-stiffness hydrogel (state II). The reverse addition of urea neutralizes the hydrogel, leading to the dissociation of the i-motif units and to the regeneration of the (**4**)/(**6**)//(**7**)/(**5**) bridges (state I). Besides, treatment of the higher-stiffness hydrogel (state I) with urea leads to the urease-catalyzed hydrolysis of the substrate and to a basic pH environment. This leads to the separation of the (**2**)/(**3**)/(**2**) T–A·T triplexes, and the resulting (**4**)/(**6**)//(**7**)/(**5**) bridges provide the sole crosslinking units, yielding a hydrogel of lower stiffness, state III (path II). The reverse treatment of the hydrogel in state III with glucose or acetylcholine neutralizes the basic condition of the hydrogel, recovering the hydrogel to state I that exhibits higher stiffness. Fig. 5b–d shows the rheometric characterization of the three biocatalyst-triggered hydrogel states and presents the reversible stiffness properties of the hydrogel. The hydrogel in state I reveals a higher stiffness, *G*′ = 177 Pa and *G*′′ = 18 Pa (curve a′ and a′′ in Fig. 5b), whereas the hydrogels in state II (*G*′ = 80 Pa, *G*′′ = 6 Pa, curve b′ and b′′) and state III (*G*′ = 106 Pa, *G*′′ = 9 Pa, curve c′ and c′′) show lower stiffness. Fig. 5c depicts the biocatalyst-driven stiffness properties upon the primary treatment of hydrogel in state I with urea, leading to the formation of a hydrogel of lower stiffness (state III). Subjecting the lower-stiffness hydrogel to glucose restores state I. The subsequent treatment of the hydrogel in state I with acetylcholine leads to lower-stiffness state II, and the following reaction of state II with urea leads to the regeneration of state I. Similarly, Fig. 5d shows the cyclic transitions of the hydrogel upon treatment with urea, acetylcholine, glucose and urea, respectively. Fig. 5e depicts the SEM images that support the stiffness properties of the different hydrogel states. While state I, panel I, shows a highly porous structure composed of dense small pores, consistent with the highly crosslinked hydrogel, the lower-stiffness hydrogels in state II (panel II) and state III (panel III) show large pores at lower densities, consistent with the lower degree of crosslinking. In addition, the control over the stiffness of the hydrogel by means of the three loaded enzymes was applied to control the multi-triggered shape-memory features, as shown in Fig. 6. The triangle-shaped, high-stiffness hydrogel is crosslinked by (**2**)/(**3**)/(**2**) triplex bridges and (**4**)/(**6**)//(**7**)/(**5**) crosslinking units. Subjecting the triangle-shaped hydrogel (state I) to urea leads to lower-stiffness, quasi-liquid state III, where the (**4**)/(**6**)//(**7**)/(**5**) bridges act as internal memory. The neutralization of the matrix upon the addition of glucose results in the memory-guided regeneration of the shaped, stiff hydrogel in state I. The subsequent acidification of the shaped hydrogel by acetylcholine leads to shapeless, low-stiffness state II that includes only the (**2**)/(**3**)/(**2**) triplex bridges as memory. The subsequent treatment of the hydrogel with urea neutralizes the matrix, resulting in the (**2**)/(**3**)/(**2**)-guided regeneration of the stiff triangle-shaped hydrogel. Finally, the treatment of the stiff neutral hydrogel with glucose re-acidifies the matrix, leading to the shapeless matrix crosslinked only by the (**2**)/(**3**)/(**2**) memory bridges.

**Fig. 7:**
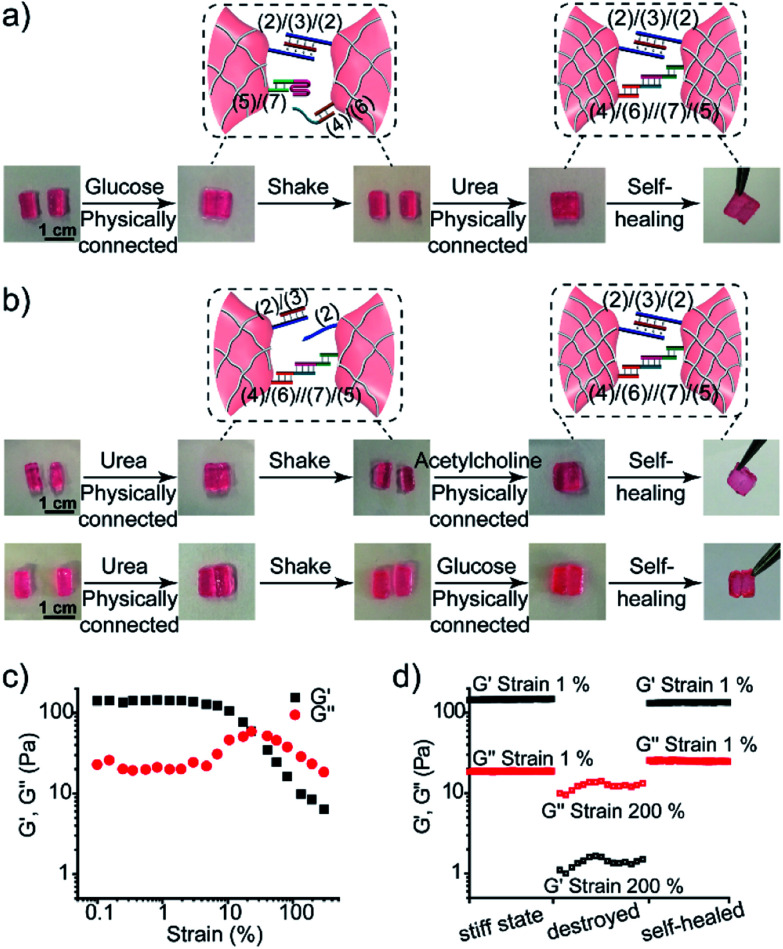
Self-healing properties of the three-enzyme-loaded hydrogel crosslinked by (**2**)/(**3**)/(**2**) and (**4**)/(**6**)//(**7**)/(**5**) bridges (*cf.*Fig. 5a): (a) self-healing of the physically joined hydrogel pieces crosslinked by (**2**)/(**3**)/(**2**) bridges *via* the cooperative formation of the healed (**4**)/(**6**)//(**7**)/(**5**) bridges. (b) Self-healing of the physically joined hydrogel pieces crosslinked by (**4**)/(**6**)//(**7**)/(**5**) bridges *via* the cooperative formation of the healed (**2**)/(**3**)/(**2**) bridges. (c) The *G*′/*G*′′ value *vs.* strain% of the stiff three-enzyme-loaded hydrogel crosslinked by (**2**)/(**3**)/(**2**) and (**4**)/(**6**)//(**7**)/(**5**) bridges. (d) Probing the self-healing of the hydrogel. Left: *G*′/*G*′′ values of the original hydrogel. Middle: *G*′/*G*′′ values of the hydrogel treated with glucose and subjected to a 200% strain. Right: The *G*′/*G*′′ values of the healed hydrogel generated upon treatment of the destructed hydrogel with urea.

The control over the stiffness properties of hydrogels by means of biocatalytic transformations was also used to develop biocatalytically driven self-healing hydrogel matrices, as shown in Fig. 7. In the first system, Fig. 7a visually depicts the self-healing process. The three-enzyme-loaded high-stiffness hydrogel, crosslinked by the T–A·T and the (**4**)/(**6**)//(**7**)/(**5**) bridges, was cut into two pieces that were treated with glucose to yield low-stiffness hydrogel pieces. Their physical connection did not lead to any self-healing, after a time interval of 1 h, and shaking the system led to the separation of the two pieces. The physical connection of the pieces, followed by the treatment with urea, led to neutralization of the hydrogel, the separation of the i-motif units, and to the cooperative stabilization of the interlinked boundary to a self-healed, intact hydrogel crosslinked by the T–A·T and (**4**)/(**6**)//(**7**)/(**5**) bridges (ESI Video 1†). In a further example, the stiff hydrogel cooperatively stabilized by the (**2**)/(**3**)/(**2**) and the (**4**)/(**6**)//(**7**)/(**5**) bridges was cut into two pieces that were treated with urea. This stimulated the separation of the T–A·T bridges, thus forming the low-stiffness hydrogel crosslinked only by the (**4**)/(**6**)//(**7**)/(**5**) bridges. The physical connection of the two pieces did not lead to self-healing. Treatment of the low-stiffness, physically connected matrices with acetylcholine or glucose neutralized the hydrogels, leading to self-healing through the formation of the stiff matrices crosslinked cooperatively by the (**2**)/(**3**)/(**2**) and (**4**)/(**6**)//(**7**)/(**5**) bridges, as shown in Fig. 7b (ESI Video 2 and 3†). The percentage of self-healing was quantitatively analyzed by strain-sweep measurements. The high-stiffness hydrogel crosslinked by the T–A·T and the (**4**)/(**6**)//(**7**)/(**5**) bridges (*cf.*Fig. 7a) reveals unchanged *G*′/*G*′′ values within a strain interval of 1–23%, as shown in Fig. 7c. The initial hydrogel showed *G*′ = 150 Pa and *G*′ = 18 Pa under 1% strain, as shown in Fig. 7d. Then, the hydrogel was treated with glucose and subjected to a 200% strain that resulted in the breakdown of the hydrogel. Subsequently, the hydrogel was treated with urea to yield the self-healed hydrogel matrix. Upon applying a 1% strain, the self-healed hydrogel matrix revealed *G*′ = 134 Pa and *G*′′ = 25 Pa as compared to the original hydrogel, implying that it recovered 89% of its original stiffness upon self-healing. Similarly, the results of the other two self-healed hydrogels described in Fig. 7b are shown in Fig. S5.†

**Fig. 8:**
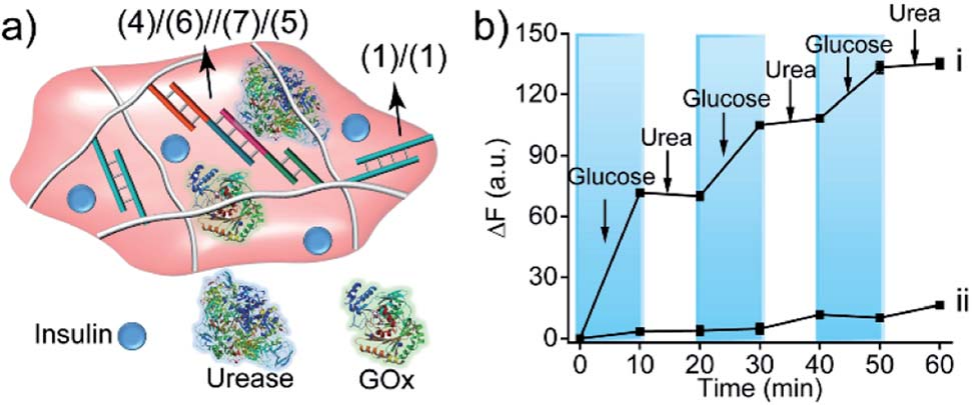
(a) Schematic structure of the GOx/urease/insulin-loaded hydrogel crosslinked by (**1**)/(**1**) and the pH-responsive (**4**)/(**6**)//(**7**)/(**5**) duplexes. (b) Curve (i) switchable release of insulin from the hydrogel upon treatment with glucose and urea. Curve (ii) release of insulin from the hydrogel in the absence of glucose and urea.

The biocatalytic control of the stiffness of the hydrogels was further applied to develop stimulus-responsive drug-loaded hydrogel matrices. Specifically, the hydrogel cooperatively crosslinked by (**1**)/(**1**) and (**4**)/(**6**)//(**7**)/(**5**) bridges was applied to demonstrate the switchable ON–OFF release of insulin. A higher-stiffness hydrogel was prepared in the presence of GOx/urease and coumarin-labeled insulin, as show in Fig. 8a (see Fig. S6† for DNA loading on polymers P_F_ and P_G_). The addition of glucose resulted in its aerobic GOx-catalyzed oxidation. The formed gluconic acid acidified the hydrogel matrix and led to the separation of (**4**)/(**6**)//(**7**)/(**5**) bridges through the reconfiguration of (**7**) into the i-motif structure, resulting in the release of insulin. Subjecting the hydrogel to urea resulted in the urease-catalyzed hydrolysis of urea and the neutralization of the hydrogel. This recovered the higher-stiffness hydrogel cooperatively stabilized by (**1**)/(**1**) and (**4**)/(**6**)//(**7**)/(**5**) bridges and blocked the release of insulin. The release of insulin could be reversibly switched between the ON/OFF states by the cyclic treatment of the hydrogel with glucose and urea, Fig. 8b(i). It should be noted that in the absence of glucose, no release of insulin could be detected (Fig. 8b(ii)).

**Fig. 9:**
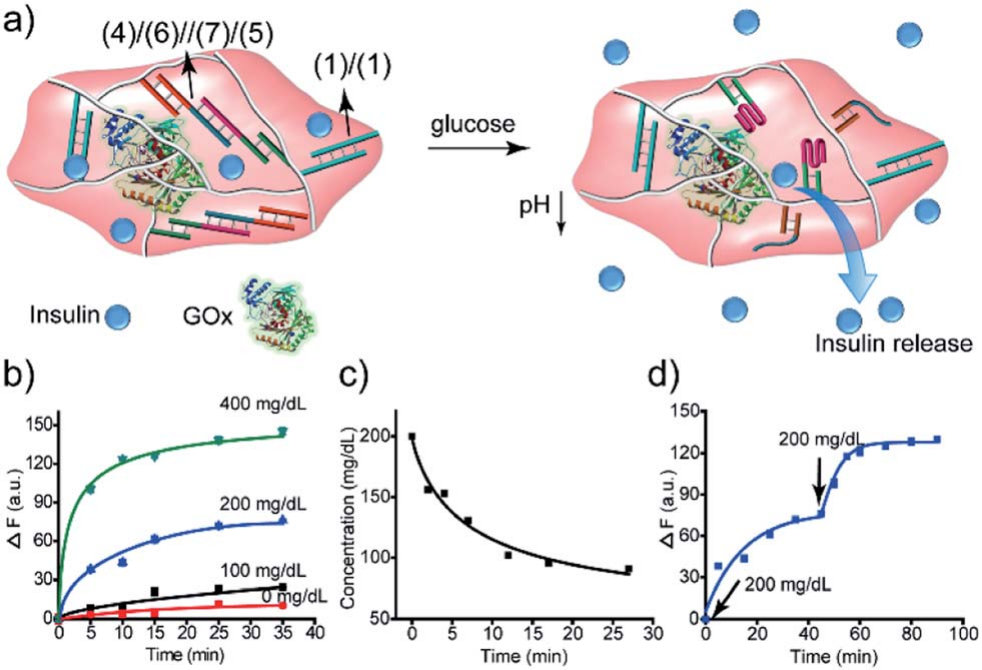
Application of the insulin-loaded glucose-responsive hydrogel crosslinked by (**1**)/(**1**) and the pH-responsive (**4**)/(**6**)//(**7**)/(**5**) supramolecular duplexes: (a) schematic structure of the stiff insulin-loaded glucose-responsive hydrogel. (b) Time-dependent release of insulin from the hydrogel in the presence of different concentrations of glucose. (c) Time-dependent depletion of glucose in a parent glucose solution (200 mg dL^−1^) treated with the GOx/catalase-loaded hydrogel. (d) Glucose-stimulated switchable release of insulin by the “Artificial Pancreas” hydrogel.

The GOx-catalyzed release of insulin from the hydrogel matrix suggests that the enzyme-responsive hydrogel could introduce a concept for tailoring an “Artificial Pancreas”, where the hydrogel acts as a carrier for the autonomous glucose-guided release of insulin. Accordingly, we argued that the GOx-stimulated pH changes in the hydrogel could be controlled by the concentrations of glucose. Thus, we integrated GOx and coumarin-labeled insulin in a higher-stiffness hydrogel matrix, as shown in Fig. 9a. Fig. 9b depicts the time-dependent release of the coumarin-labeled insulin from the hydrogel, in the presence of variable concentrations of glucose. No significant leakage or release of insulin was observed in the absence of glucose. In the presence of 100 mg dL^−1^ glucose (normal glucose level), a negligible release of insulin was observed, implying that at these concentrations of glucose, the pH changes in the matrix are too low to change the stiffness of the hydrogel and induce the release of insulin. At a higher glucose concentration, 200 mg dL^−1^ or 400 mg dL^−1^, effective release of insulin was observed. Of particular interest, is the insulin release profile at an elevated glucose concentration of 200 mg dL^−1^ that is relevant for diabetes control. The release of insulin proceeded for *ca.* 30 min, reaching a saturated state due to a decrease in glucose concentration consumed in the processes. Indeed, monitoring the glucose concentrations with a glucometer revealed a rapid decrease of glucose in the hydrogel (Fig. 9c). Subjecting the glucose-responsive GOx/insulin-loaded hydrogel that reached the saturation insulin-release level to an additional high concentration level of glucose (200 mg dL^−1^) switched-on the release of insulin (Fig. 9d), implying that the hydrogel exhibits the glucose-controlled switchable release functionality for autonomous operation. The glucose-stimulated controlled release of insulin, particularly for glucose concentrations relevant for controlling diabetes, suggests that such hydrogel matrices could act as artificial pancreas patches for the autonomous release of insulin (see also the Conclusion paragraph). In fact, different previous studies have discussed the possibilities of developing artificial pancreas systems for the autonomous switchable release of insulin.^60–62^ Nonetheless, these systems operated at irrelevant concentrations of glucose or the switching ON/OFF release time-intervals of insulin were inappropriate for controlling diabetes. The formation of the toxic H_2_O_2_, a ROS-generated product, accompanying the aerobic GOx-catalyzed oxidation of glucose, needs to be addressed to support the potential utility of the system. To overcome this limitation, we co-immobilized catalase in the GOx/insulin-loaded hydrogel. Catalase induced a catalyzed disproportionation of H_2_O_2_ into O_2_ and H_2_O, and, thus, the biocatalytically generated H_2_O_2_ in the glucose-responsive hydrogel was anticipated to be degraded. Indeed, Fig. S7† and the accompanying discussion revealed that all H_2_O_2_ generated by the glucose-responsive hydrogel that includes GOx/catalase/insulin loads was degraded. A further aspect to consider relates to the stability of the DNA-based hydrogel towards DNase. Fig. S9† and the accompanying discussion show the performance of GOx-loaded hydrogel, cooperatively crosslinked by (**1**)/(**1**) and (**4**)/(**6**)//(**7**)/(**5**) bridges, treated with DNase at different time-intervals. We did not observe a decrease in the GOx-activity after a time-interval of 3 days, implying that no GOx release and no hydrogel degradation of the hydrogel occurred within this time-interval. Presumably, the hydrogel matrix protects the DNA-bridging units from being digested by DNase within this time-interval. It should be noted that the enzymes incorporated in the hydrogel matrices retained >90% of their activity as compared to the native enzymes.

## Conclusions

The present study has integrated enzymes in DNA-based hydrogels as a means to control their stiffness. Two or three different enzymes were loaded in nucleic acid bridged polyacrylamide hydrogels. The biocatalysts inducing the acidification of the hydrogels were glucose oxidase (GOx) and acetylcholine esterase (AchE) and the enzyme that introduced basic conditions into the hydrogels was urease. The hydrogels were cooperatively crosslinked by two kinds of pH-responsive bridges, where T–A·T bridging units were dissociated under basic conditions generated by the urease-catalyzed decomposition of urea, and the duplex nucleic acids were separated by their reconfiguration into i-motif structures under acidic conditions, generated by the biocatalytic aerobic GOx-catalyzed oxidation of glucose to gluconic acid and H_2_O_2_ or by the AchE-catalyzed hydrolysis of acetylcholine to acetic acid and choline. By the selective pH-stimulated separation of the respective nucleic acid crosslinking units, the control over the stiffness of the hydrogels was demonstrated. In fact, other enzymes, *e.g.*, phosphatase or carbonic anhydrase, could be also used to control the pH conditions in hydrogels. The enzyme-guided switchable control over the stiffness of hydrogels provides novel methods to develop biocatalytically driven shape-memory and self-healing matrices. In addition, the biocatalytically switched stiffness properties of the hydrogels were used for the substrate-triggered controlled drug release. Specifically, the glucose-triggered control over the stiffness of the GOx/insulin-loaded hydrogel and the glucose dose-controlled release of insulin provide means for the autonomous regulation of glucose levels by the released insulin. One could envision the deposition of the GOx/catalase/insulin-loaded hydrogel, described in this paper, on a capillary microneedle array as a functional device acting as an artificial pancreas. The hydrogel/microneedle array may act as a patch on the skin for the autonomous sensing of glucose in subcutaneous fluids followed by the autonomous release of insulin and its sequestered delivery to the body.^63^

## Conflicts of interest

There are no conflicts to declare.

## Supplementary Material

SC-011-D0SC01319F-s001

SC-011-D0SC01319F-s002

SC-011-D0SC01319F-s003

SC-011-D0SC01319F-s004
