# Supplementary material for: Biocatalytic reversible control of the stiffness of DNA-modified responsive hydrogels: applications in shape-memory, self-healing and autonomous controlled release of insulin
Source: Chem Sci. 2020 Apr 14;11(17):4516–24. doi: 10.1039/d0sc01319f (PMC8159436; doi:10.1039/d0sc01319f)
Supplement: SC-011-D0SC01319F-s001 [file SC-011-D0SC01319F-s001.pdf]

Electronic Supplementary Material (ESI) for Chemical Science.
This journal is © The Royal Society of Chemistry 2020

Biocatalytic Reversible Control of the Stiffness of DNA-Modified Responsive Hydrogels: Applications as Shape-Memory, Self-Healing and Autonomous Controlled Release of Insulin

Chen Wang,‡, [a] Amit Fischer,‡, [a] Avner Ehrlich,[b] Yaakov Nahmias,[b] Itamar Willner*[a]

Table of Contents

Experimental Procedures 3
Chemicals 3
Measurement 3
Synthesis of polymers 3
Polymer P_A 3
Polymer P_B 3
Polymer P_C 3
Polymer P_D 4
Polymer P_E 4
Synthesis of coumarin-insulin 4
Synthesis of GOx-FITC 4
Synthesis of urease-RhB 4
Synthesis of AchE-FITC 4
Synthesis of Catalase-RhB 4
Preparation of hydrogels 4
Preparation of hydrogel crosslinked by (**1**)/(**1**) and (**2**)/(**3**)/(**2**) bridges 4
Preparation of hydrogel crosslinked by (**1**)/(**1**) and (**4**)/(**6**)//(**7**)/(**5**) bridges 5
Preparation of hydrogel crosslinked by (**2**)/(**3**)/(**2**) and (**4**)/(**6**)//(**7**)/(**5**) bridges 5
Rheometric experiments 5
Shape-memory experiments 5
Self-healing experiments 5
Release of coumarin-insulin from the hydrogel crosslinked by (**1**)/(**1**) and (**4**)/(**6**)//(**7**)/(**5**) bridges 6
Calculation of the loaded amounts of enzymes and insulin 6
pH changes of the HEPES buffer before and after adding respective triggers into the hydrogel 6
The performance of GOx-loaded hydrogel crosslinked by (**1**)/(**1**) and (**4**)/(**6**)//(**7**)/(**5**) bridges treated with DNAase 7
Results and Discussion 7
References 15

Experimental Procedures

Chemicals

Magnesium chloride, 4-(2-hydroxyethyl)piperazine-1-ethanesulfonic acid sodium salt (HEPES), phosphate buffer (sodium phosphate dibasic, sodium phosphate monobasic), acrylamide (AAm), 2-hydroxy-4′-(2-hydroxyethoxy)-2-methylpropiophenone (irgacure D-2959), glucose (glu), urea, acetylcholine (ac), glucose oxidase (GOx, 187300 units/g), urease (15000-50000 units/g), acetylcholinesterase (AchE, 658 units/g), catalase (11895 units/mg, 24 mg/mL), Amplex Red, 7-hydroxycoumarin-3-carboxylic acid N-succinimidyl ester, fluorescein isothiocyanate isomer I (FITC), Rhodamine B isothiocyanate (RhB), insulin, ethylenediaminetetraacetic acid disodium salt dihydrate (EDTA), Gel Red were purchased from Sigma-Aldrich. Texas Red-dextran (M_w = 70K Da) was purchased from Life Technologies Corporation (USA). Desalted nucleic acid strands were purchased from Integrated DNA Technologies Inc. (Coralville, IA). Ultrapure water purified by a NANOpure Diamond instrument (Barnstead International, Dubuque, IA, USA).

No.	DNA sequence (5’→3’)
(**1**)	5′-acrydite-AAATTCGCGCGCGAA-3’
(**2**)	5′-acrydite-TTC TTT TCT TTT CTT TTC TT-3′
(**3**)	5′-AAG AAA AGA AAA GAA AAG AA-3′
(**4**)	5’-acrydite-AAACCAACCAACCAATTC-3’
(**5**)	5’-acrydite-AAAAGAGAGAGAGATGAC-3’
(**6**)	5’-G TTA GTG TTA GTG TTA GTGAATTGGTTGGTTGG-3'
(**7**)	5'-T CCC TAA CCC TAA CCC TAA CCC GTCATCTCTCTCTCTAAA-3'

Measurement

The absorbance spectra were recorded by a UV-2450 spectrophotometer (Shimadzu). SEM images were taken by using High Resolution Scanning Electron Microscope Sirion (setting: 5 kV). The samples were first freeze-dried and placed on silica slides, coated with gold-palladium by using Sputter Coater. The fluorescence spectra were recorded by a Varian Cary Eclipse spectrofluorometer with a slit width of 5 nm. The stiffness properties of hydrogels were measured with a HAAKE MARS III rheometer (Thermo Scientific). The glucose concentrations were measured with glucometer Accutrend plus kit mg/dl with Accutrend Glucose II 25 STR strips (Roche).

Synthesis of polymers

Polymer P_A

AAm (6 mg), DNA (**1**) (5 mM, 27 μL), DNA (**2**) (5 mM, 81 μL) and irgacure D-2959 (0.4 M, 2.1 μL) were mixed and bubbled with N_2 for 15 min, followed by UV irradiation for 10 min to form the copolymer P_A. After polymerization, the unreacted monomers and initiator were washed away using an Amicon filter (5 KDa MWCO) for three times (7000 r/min, 15 min) and freeze-dried.

Polymer P_B

AAm (6 mg), DNA (**1**) (5 mM, 27 μL), DNA (**4**) (5 mM, 81 μL) and irgacure D-2959 (0.4 M, 2.1 μL) were mixed and bubbled with N_2 for 15 min, followed by the same procedure as described in the synthesis of polymer P_A.

Polymer P_C

AAm (6 mg), DNA (**1**) (5 mM, 27 μL), DNA (**5**) (5 mM, 81 μL) and irgacure D-2959 (0.4 M, 2.1 μL) were mixed and bubbled with N_2 for 15 min, followed by the same procedure as described in the synthesis of polymer P_A.

Polymer P_D

AAm (6 mg), DNA (**2**) (5 mM, 81 μL), DNA (**4**) (5 mM, 81 μL) and irgacure D-2959 (0.4 M, 2.1 μL) were mixed and bubbled with N_2 for 15 min, followed by the same procedure as described in the synthesis of polymer P_A.

Polymer P_E

AAm (6 mg), DNA (**2**) (5 mM, 81 μL), DNA (**5**) (5 mM, 81 μL) and irgacure D-2959 (0.4 M, 2.1 μL) were mixed and bubbled with N_2 for 15 min, followed by the same procedure as described in the synthesis of polymer P_A.

Synthesis of coumarin-insulin

The synthesis of coumarin-insulin was according to previous literature.[1] Insulin (5 mg) and 7-hydroxycoumarin-3-carboxylic acid N-succinimidyl ester (16 mg dissolved in 300 μL acetone) were dissolved in HEPES buffer (100 mM, pH 7) that contained EDTA (200 μM) to prevent aggregation of insulin. The reaction was mixed in the dark for 8 h, and then the mixture was washed with an Amicon filter (3 KDa MWCO) for three times (8000 r/min, 10 min).

Synthesis of GOx-FITC

FITC (13 mg) was dissolved in acetone (100 μL), GOx (10 mg) was dissolved in phosphate buffer (100 mM, pH 7) that contained EDTA (200 μM). Mixing the two solutions and the reaction was kept in dark for 8 h and then the mixture was washed with an Amicon filter (30 KDa MWCO) for three times (8000 r/min, 10 min).

Synthesis of urease-RhB

Urease (5 mg) and RhB (4.7 mg in methanol) were mixed in phosphate buffer (100 mM, pH 7) that contained EDTA (200 μM). The reaction was mixed in the dark for 8 h, and then the mixture was washed with an Amicon filter (30 KDa MWCO) for three times (8000 r/min, 10 min).

Synthesis of AchE-FITC

FITC (2.8 mg) was dissolved in acetone (100 μL), AchE (1 mg) was dissolved in phosphate buffer (100 mM, pH 7) that contained EDTA (200 μM). Mixing the two solutions and the reaction was kept in dark for 8 h and then the mixture was washed with an Amicon filter (30 KDa MWCO) for three times (8000 r/min, 10 min).

Synthesis of Catalase-RhB

Catalase (1 mL) and RhB (10 mg in methanol) were mixed in phosphate buffer (100 mM, pH 7) that contained EDTA (200 μM). The reaction was mixed in the dark for 8 h, and then the mixture was washed with an Amicon filter (30 KDa MWCO) for three times (8000 r/min, 10 min).

Preparation of hydrogels

*Preparation of hydrogel crosslinked by (**1**)/(**1**) and (**2**)/(**3**)/(**2**) bridges*

Polymer P_A (4 mg) and DNA (**3**) (5mM, 12.8 μL) was mixed in 110 μL of 10 mM HEPES buffer (100 mM Mg^{2+}, pH 7.4) and heated for 10 min, followed by cooling down in ice bath. Then GOx (50 mg/mL, 3.2 μL, 30 units)/urease (250 mg/mL, 1.6 μL, 30 units) or AchE

(15 mg/mL, 3 μL, 30 units)/urease (250 mg/mL, 1.6 μL, 30 units) were added, together with 10 μL of HEPES buffer, into the solution and kept at 4 °C overnight to form the hydrogel (3 wt%). The hydrogel was stained with Texas Red-dextran (6.5 mg/mL, 2 μL).

*Preparation of hydrogel crosslinked by (**1**)/(**1**) and (**4**)/(**6**)//(**7**)/(**5**) bridges*

Polymer P_B (2 mg) was dissolved in HEPES buffer (10 mM, 100 mM Mg^{2+}, pH 7.4, 50 μL) and mixed with DNA (**6**) (5 mM, 23 μL) to form the polymer P_B'. Polymer P_C (2 mg) was dissolved in HEPES (10 mM, 100 mM Mg^{2+}, pH 7.4, 40 μL) and mixed with DNA (**7**) (5 mM, 12.4 μL) to form the polymer P_C'. Mixing the solutions of P_B' and P_C' and heating until the polymers were completely dissolved, followed by cooling down in ice bath. Then GOx (50 mg/mL, 3.2 μL, 30 units)/urease (250 mg/mL, 1.6 μL, 30 units) or AchE (15 mg/mL, 3 μL, 30 units)/urease (250 mg/mL, 1.6 μL, 30 units) were added, together with 10 μL of HEPES buffer and Gel Red (10 mM, 1 μL), into the solution and kept at 4 °C overnight to form the hydrogel (3 wt%).

*Preparation of hydrogel crosslinked by (**2**)/(**3**)/(**2**) and (**4**)/(**6**)//(**7**)/(**5**) bridges*

Polymer P_D (1.2 mg) was dissolved in HEPES buffer (10 mM, 100 mM Mg^{2+}, pH 7.4, 40 μL) and mixed with DNA (**6**) (5 mM, 9.8 μL) to form the polymer P_D'. Polymer P_E (2 mg) was dissolved in HEPES (10 mM, 100 mM Mg^{2+}, pH 7.4, 50 μL) and mixed with DNA (**7**) (5 mM, 4.4 μL) to form the polymer P_E'. Mixing the solutions of P_D' and P_E' with DNA (**3**) (5 mM, 14 μL) and heating until the polymers were completely dissolved, followed by cooling down in ice bath. Then GOx (50 mg/mL, 3.2 μL, 30 units)/urease (250 mg/mL, 1.6 μL, 30 units)/AchE (15 mg/mL, 3 μL, 30 units) were added, together with 10 μL of HEPES buffer and Gel Red (10 mM, 1 μL), into the solution and kept at 4 °C overnight to form the hydrogel (3 wt%).

Rheometric experiments

In rheometric experiments, the hydrogels were prepared with a volume of 150 μL of the polymer solution (3 wt%). The strain-dependent changes of the storage (G') and loss (G'') moduli were measured at 20 °C with a gap distance of 0.3 mm, 1 Hz frequency and strain sweeps of amplitude 0.1%-300% (*cf.* Figure 7c). The frequency-dependent changes of the storage (G') and loss (G'') moduli were measured at 20 °C with a gap distance of 0.3 mm, 1% strain (Figure S8). The time-dependent stiffness of hydrogels was measured at 20 °C with a gap distance of 0.3 mm, 1% strain and 1 Hz frequency.

Shape-memory experiments

In shape-memory experiments, the respective hydrogel was prepared with a volume of 80 μL (3 wt%) in a triangle mold. The solution of polymers was mixed in an eppendorf tube and heated until the polymers were completely dissolved, followed by transferring the solution into the mold. Then the mold was cooled down in ice bath, then adding the respective amounts of enzymes into the mold. After being kept at 4 °C overnight to form the hydrogel, the hydrogel was extruded from the mold as a triangle shape and treated with different triggers (glucose, acetylcholine or urea, final concentration of the trigger was 10 mM) for a time interval of 60 min, to trigger the switchable transitions of the hydrogel. The triangle-shaped hydrogels were stained with Gel Red or Texas Red-dextran.

Self-healing experiments

In self-healing experiments, the hydrogels were prepared with a volume of 80 μL (3 wt%) in a cubic mode. Respective polymers were mixed in an eppendorf tube and heated until the polymers were completely dissolved, followed by transferring the solution into the mold. Then the mold was cooled down in ice bath, then adding the respective amounts of enzymes into the mold. After being kept at 4 °C overnight to form the hydrogel. The hydrogel was extruded from the mold as a cubic shape and cut into two pieces, followed by treating with different triggers (glucose, acetylcholine or urea, final concentration of the trigger was 10 mM) for a time interval of 60 min, to trigger the switchable transitions and self-healing behavior of the hydrogel. The hydrogels were stained with Gel Red.

Release of coumarin-insulin from the hydrogel crosslinked by (1)/(1) and (4)/(6)//(7)/(5) bridges

AAm (3 mg), DNA (**1**) (5 mM, 40.5 μL), DNA (**4**) (5 mM, 13.5 μL) and irgacure D-2959 (0.4 M, 1.05 μL) were mixed and bubbled with N_2 for 15 min, followed by UV irradiation for 10 min to form the copolymer P_F. After polymerization, the unreacted monomers and initiator were washed away using an Amicon filter (5 KDa MWCO) for three times (7000 r/min, 15 min) and freeze-dried.
AAm (3 mg), DNA (**1**) (5 mM, 40.5 μL), DNA (**5**) (5 mM, 13.5 μL) and irgacure D-2959 (0.4 M, 1.05 μL) were mixed and bubbled with N_2 for 15 min, followed by UV irradiation for 10 min to form the copolymer P_G. After polymerization, the unreacted monomers and initiator were washed away using an Amicon filter (5 KDa MWCO) for three times (7000 r/min, 15 min) and freeze-dried.
Mixing P_F (1 mg) and DNA (**6**) (5mM, 3.2 μL) in 40 μL of HEPES buffer forms solution A, mixing P_G (1 mg) and DNA (**7**) (5 mM, 3 μL) in 40 μL of HEPES buffer forms solution B. Solutions A and B were mixed and heated until the solution completely dissolved, followed by adding 10 μL of coumarin-insulin stock solution (16 mM),10 μL of H_2O and heated for additional 1-3 min, later on the hydrogels were cooled down in ice bath. After cooling down, GOx (50 mg/mL, 3.2 μL)/urease (250 mg/mL, 1.6 μL) and H_2O (5 μL) were added and kept at 4 °C overnight to form the hydrogels. Then the hydrogels were used for release of insulin in the presence of different concentration of glucose.
For the time-dependent release of coumarin-insulin, the hydrogels were treated with different concentrations of glucose (0, 100, 200, 400 mg/dL), respectively, to trigger the release of coumarin-insulin. For the ON-OFF release, the hydrogel was treated with glucose (200 mg/dL) for a time-interval of 10 min to trigger the release, followed by treating with urea (200 mg/dL) for a time-interval of 10 min to stop release.
During the time-dependent release in the presence of 200 mg/dL of glucose, the decrease of glucose was monitored by a glucose meter. It should be noted that this sample should be mixed with catalase to consume H_2O_2.

Calculation of the loaded amounts of enzymes and insulin

In order to calculate the loading amounts of insulin, GOx, urease, AchE, the fluorophore-labelled insulin and fluorophore-labelled enzymes were mixed with the polymer solution before formation of hydrogels (see the details of preparation in experimental section: preparation of hydrogels). Then the hydrogels were washed with HEPES buffer three times, and the fluorescence of the washing buffer was measured (Figure S2). By comparation of the fluorescence of the loads before the formation of hydrogels with the fluorescence of the washing buffer, the loading amount of insulin could be calculated according to the calibration curve in different concentrations (Figure S10). The loading amounts were calculated to be: insulin (85.3%), GOx (77.2%, 23 units), urease (92.4%, 28 units) and AchE (90%, 27 units).

pH changes of the HEPES buffer before and after adding respective triggers into the hydrogel

Table S1. pH changes induced by different concentrations of GOx before and after adding glucose in HEPES buffer

GOx units in HEPES buffer	Initial pH	pH after adding glucose
2	7.40	6.41
10	7.40	6.04
30	7.40	5.42

Table S2. pH changes induced by different concentrations of urease before and after adding urea in HEPES buffer

Urease units in HEPES buffer	Initial pH	pH after adding urea
2	7.40	9.64
10	7.40	9.66
30	7.40	9.91

Table S3. pH changes induced by different concentrations of AchE before and after adding acetylcholine in HEPES buffer

AchE units in HEPES buffer	Initial pH	pH after adding acetylcholine
2	7.40	5.20
10	7.40	4.52
30	7.40	4.42

Table S4. pH changes induced by different concentrations of loaded enzymes in hydrogels before and after adding triggers in HEPES buffer

Enzymes loaded in hydrogels	pH changes upon the sequential addition of triggers
GOx 23 units<br>Urease 28 units	7.40 $\xrightarrow{glucose}$ 5.44 $\xrightarrow{urea}$ 7.35 $\xrightarrow{glucose}$ 5.49
GOx 23 units<br>Urease 28 units	7.40 $\xrightarrow{urea}$ 9.87 $\xrightarrow{glucose}$ 7.35 $\xrightarrow{urea}$ 9.58
AchE 27 units<br>Urease 28 units	7.40 $\xrightarrow{acetylcholine}$ 4.48 $\xrightarrow{urea}$ 7.38 $\xrightarrow{acetylcholine}$ 5.28
AchE 27 units<br>Urease 28 units	7.40 $\xrightarrow{urea}$ 9.68 $\xrightarrow{acetylcholine}$ 7.51 $\xrightarrow{urea}$ 9.21

The performance of GOx-loaded hydrogel crosslinked by (1)/(1) and (4)/(6)//(7)/(5) bridges treated with DNAase

The performance of GOx-loaded hydrogel cooperatively crosslinked by (**1**)/(**1**) and (**4**)/(**6**)//(**7**)/(**5**) bridges treated with DNAase (0.1 units/mL) for different time-intervals (30 min, 1 day, 2 day, and 3 day). After different time intervals, the hydrogel was washed three times and the GOx activity in the hydrogel was probed by adding glucose (2.5 M, 1 µL), Amplex red (20 mM, 1 µL) and HRP (1mg/mL, 1 µL). The GOx activity was not affected as compared to the diffusional enzyme mixture in buffer solution (identical enzyme concentration) (Figure S9), implying that no GOx release and no hydrogel degradation proceed within this time-interval. Presumably, the hydrogel matrix protects the DNA-bridging units from being digested by DNAase with this time-interval.

Results and Discussion

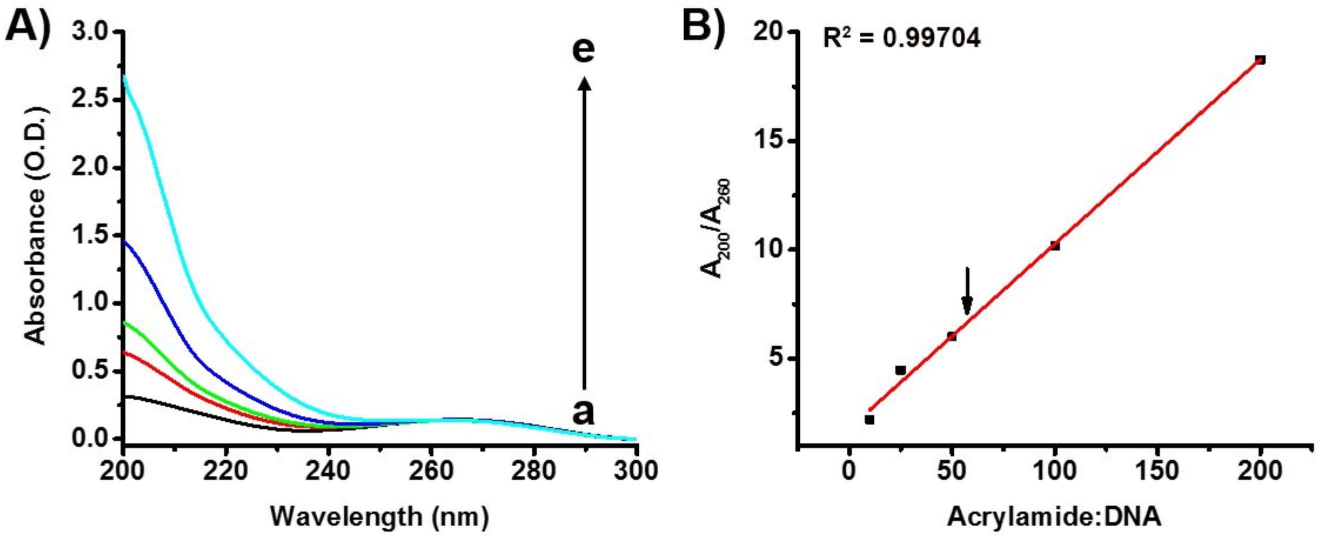

Figure S1. (A) Determination of the loading of the nucleic acid (**1**) and (**2**) on P_A: Absorption spectra of different concentrations of acrylamide monomer in the presence of a constant concentration of acrydite modified nucleic acid (**2**), 0.75µM, and (**1**), 0.25µM: (a) 10 µM (b) 25 µM (c) 50 µM (d) 100 µM (e) 200 µM. (B) Calibration curve corresponding to the absorbance ratio A_{200nm}/A_{260nm} as a function of acrylamide/DNA ratio. The arrow indicates the loading of DNA on P_A, corresponding to acrylamide: acrydite modified nucleic acid (**1**) + (**2**) = 58:1.

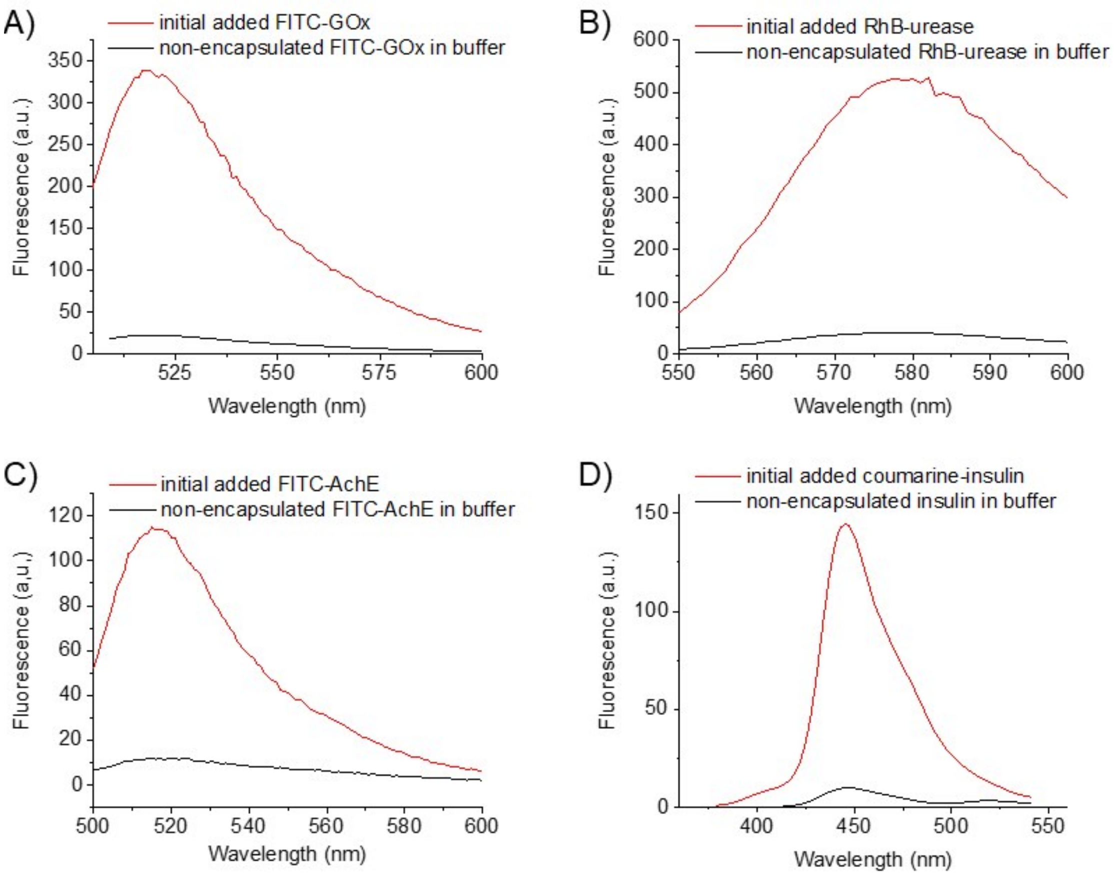

Figure S2. (A) The fluorescence spectrum of: initial added FITC-labelled GOx and the non-encapsulated GOx in HEPES buffer after washing the hydrogel three times. (B) The fluorescence spectrum of: initial added RhB-labelled urease and the non-encapsulated urease in HEPES buffer after washing the hydrogel three times. (C) The fluorescence spectrum of: initial added FITC-labelled AchE and the non-encapsulated AchE in HEPES buffer after washing the hydrogel three times. (D) The fluorescence spectrum of: initial added coumarin-labelled insulin and the non-encapsulated insulin in HEPES buffer after washing the hydrogel three times.

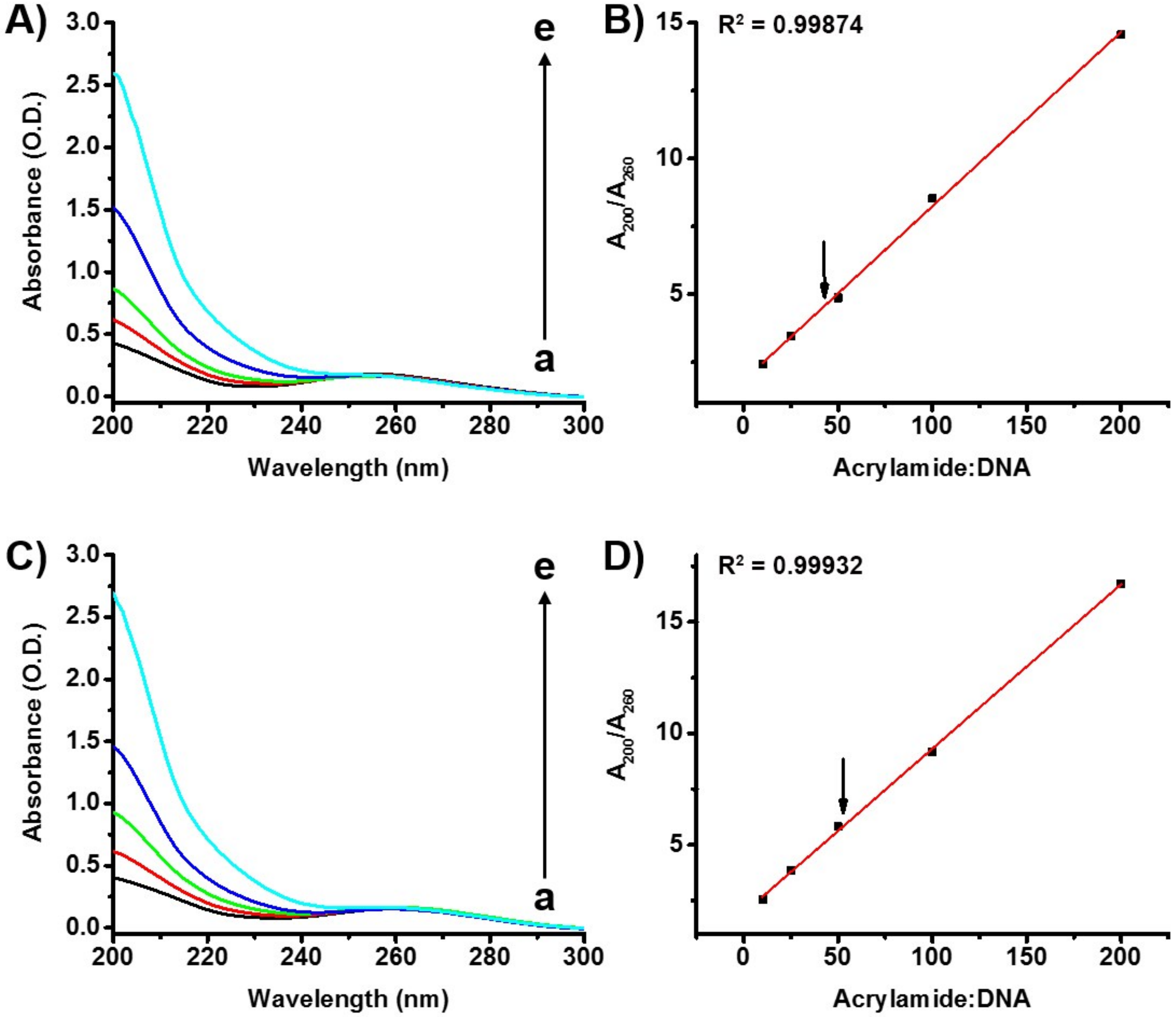

Figure S3. (A) Determination of the loading of the nucleic acid (**1**) and (**4**) on P_B: Absorption spectra of different concentrations of acrylamide monomer in the presence of a constant concentration of acrydite modified nucleic acid (**4**), 0.75μM, and (**1**), 0.25 μM: (a) 10 μM (b) 25 μM (c) 50 μM (d) 100 μM (e) 200 μM. (B) Calibration curve corresponding to the absorbance ratio A_{200nm}/A_{260nm} as a function of acrylamide/DNA ratio. The arrow indicates the loading of DNA on P_B, corresponding to acrylamide: acrydite modified nucleic acid (**1**) + (**4**) = 40:1. (C) Determination of the loading of the nucleic acid (**1**) and (**5**) on P_C: Absorption spectra of different concentrations of acrylamide monomer in the presence of a constant concentration of acrydite modified nucleic acid (**5**), 0.75μM, and (**1**), 0.25μM: (a) 10 μM (b) 25 μM (c) 50 μM (d) 100 μM (e) 200 μM. (D) Calibration curve corresponding to the absorbance ratio A_{200nm}/A_{260nm} as a function of acrylamide/DNA ratio. The arrow indicates the loading of DNA on P_C, corresponding to acrylamide: acrydite modified nucleic acid (**1**) + (**5**) = 53:1.

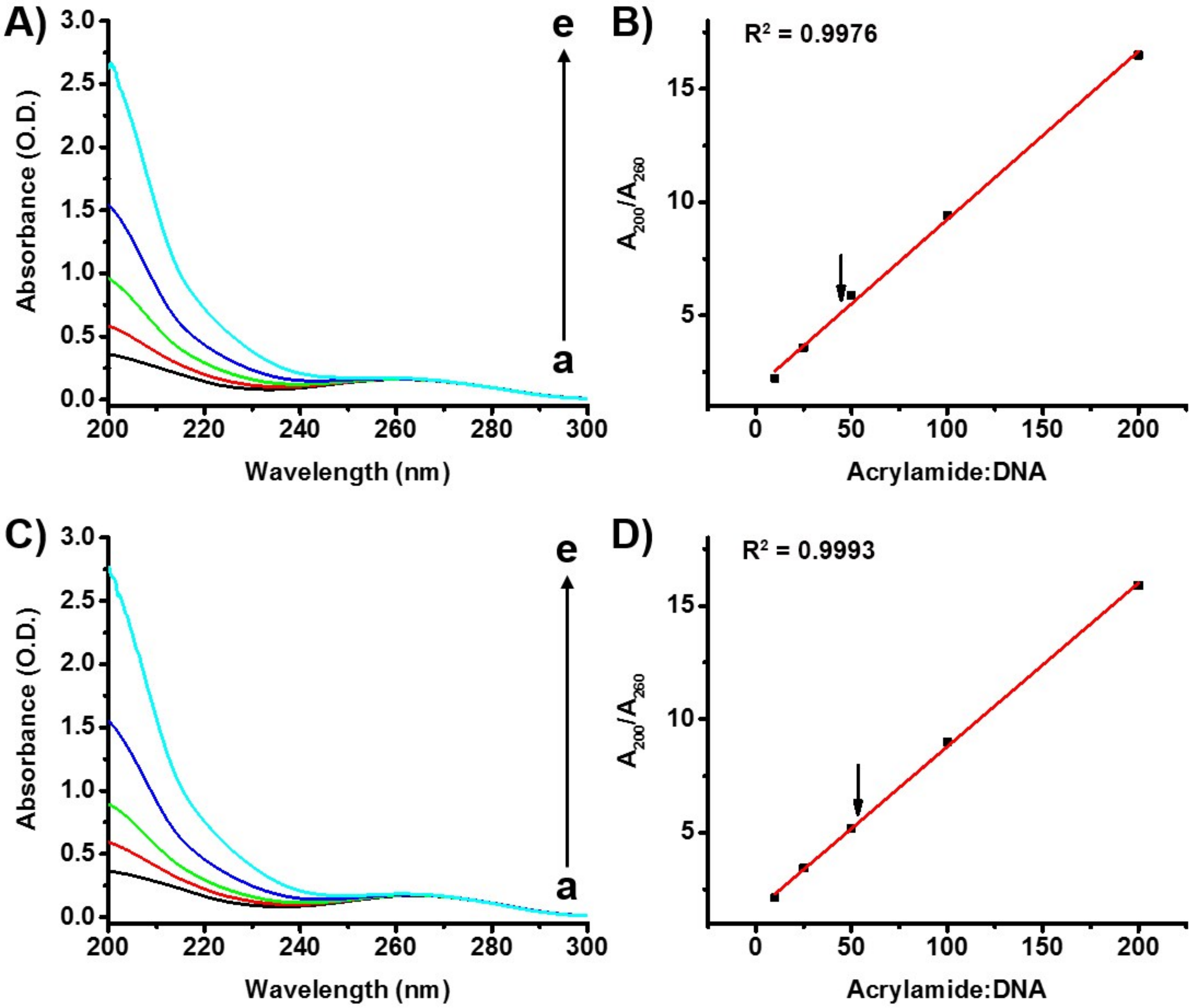

Figure S4. (A) Determination of the loading of the nucleic acid (**2**) and (**4**) on P_D: Absorption spectra of different concentrations of acrylamide monomer in the presence of a constant concentration of acrydite modified nucleic acid (**4**), 0.5μM, and (**2**), 0.5μM: (a) 10 μM (b) 25 μM (c) 50 μM (d) 100 μM (e) 200 μM. (B) Calibration curve corresponding to the absorbance ratio A_{200nm}/A_{260nm} as a function of acrylamide/DNA ratio. The arrow indicates the loading of DNA on P_D, corresponding to acrylamide: acrydite modified nucleic acid (**2**) + (**4**) = 45:1. (C) Determination of the loading of the nucleic acid (**2**) and (**5**) on P_E: Absorption spectra of different concentrations of acrylamide monomer in the presence of a constant concentration of acrydite modified nucleic acid (**5**), 0.5μM, and (**2**), 0.5μM: (a) 10 μM (b) 25 μM (c) 50 μM (d) 100 μM (e) 200 μM. (D) Calibration curve corresponding to the absorbance ratio A_{200nm}/A_{260nm} as a function of acrylamide/DNA ratio. The arrow indicates the loading of DNA on P_E, corresponding to acrylamide: acrydite modified nucleic acid (**2**) + (**5**) = 54:1.

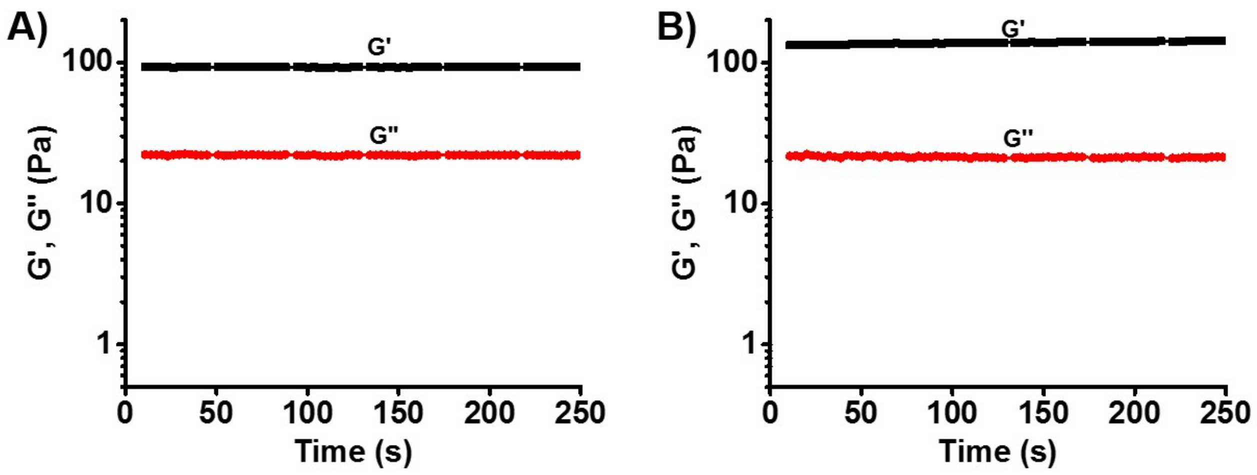

Figure S5. The stiffness of self-healed hydrogel including three enzymes GOx/AchE/Urease upon treatment with urea/acetylcholine (A) or urea/glucose (B).

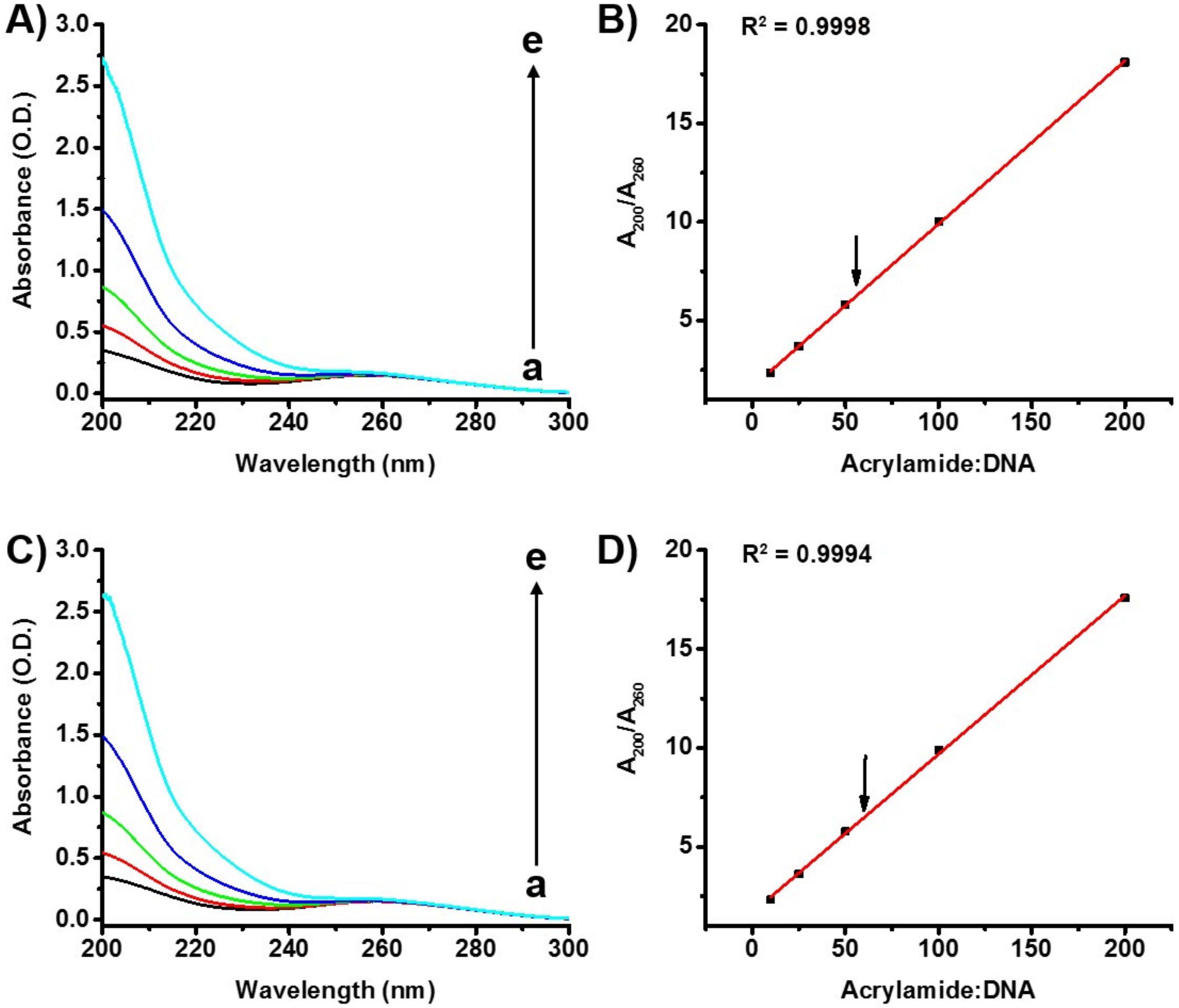

Figure S6. (A) Determination of the loading of the nucleic acid (**1**) and (**4**) on P_F: Absorption spectra of different concentrations of acrylamide monomer in the presence of a constant concentration of acrydite modified nucleic acid (**4**), 0.25μM, and (**1**), 0.75μM: (a) 10 μM (b) 25 μM (c) 50 μM (d) 100 μM (e) 200 μM. (B) Calibration curve corresponding to the absorbance ratio A_{200nm}/A_{260nm} as a function of acrylamide/DNA ratio. The arrow indicates the loading of DNA on P_F, corresponding to acrylamide: acrydite modified nucleic acid (**1**) + (**4**) = 56:1. (C) Determination of the loading of the nucleic acid (**2**) and (**5**) on P_G: Absorption spectra of different concentrations of acrylamide monomer in the presence of a constant concentration of acrydite modified nucleic acid (**5**), 0.25μM, and (**1**), 0.75μM: (a) 10 μM (b) 25 μM (c) 50 μM (d) 100 μM (e) 200 μM. (D) Calibration curve corresponding to the absorbance ratio A_{200nm}/A_{260nm} as a function of acrylamide/DNA ratio. The arrow indicates the loading of DNA on P_G, corresponding to acrylamide: acrydite modified nucleic acid (**1**) + (**5**) = 62:1.

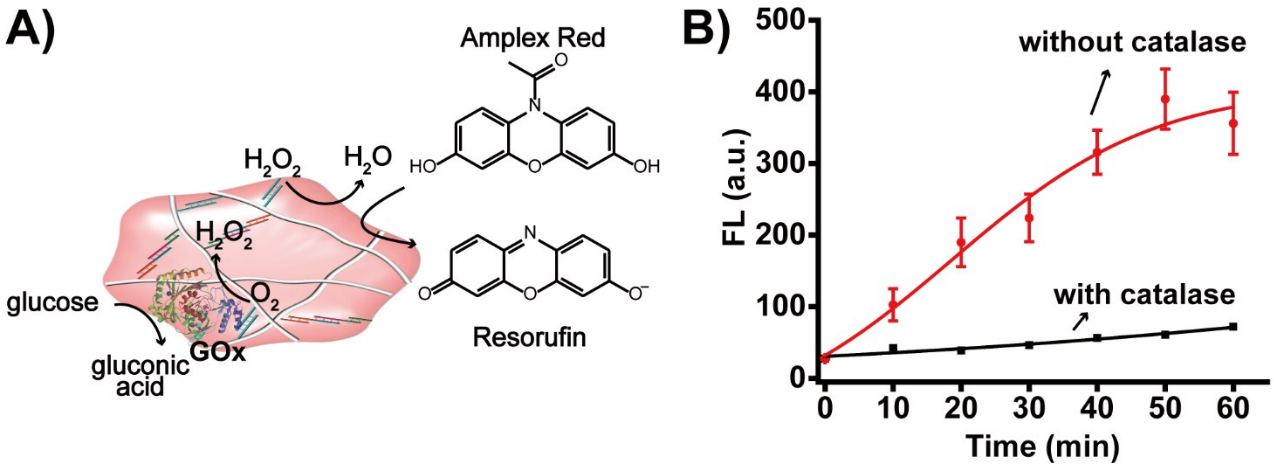

Figure S7. (A) Operation of a cascade within the hydrogel. The aerobic oxidation of glucose yields gluconic acid and H_2O_2. The generated H_2O_2 acts as a substrate that catalyzes the oxidation of Amplex Red to Resorufin. The latter product provides the fluorescence readout signal for the biocatalytic cascade. (B) Time-dependent fluorescence changes generated in the presence of catalase and in the absence of catalase.

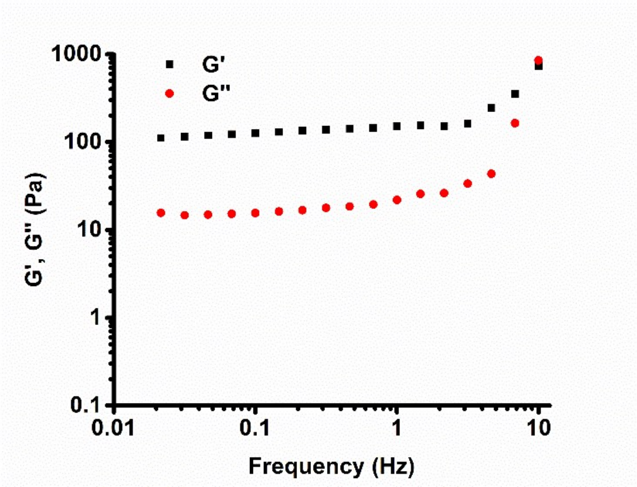

Figure S8. Frequency dependence of G', G'' of the hydrogel including three enzymes GOx/AchE/Urease.

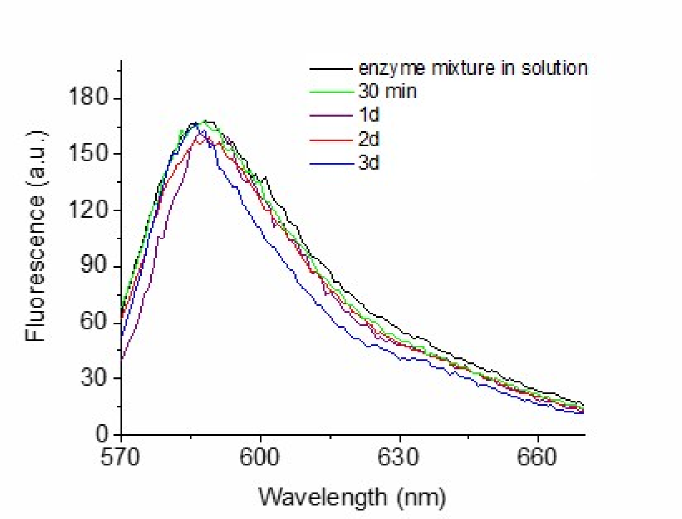

Figure S9. The performance of GOx-loaded hydrogel cooperatively crosslinked by (1)/(1) and (4)/(6)//(7)/(5) bridges treated with DNAase (0.1 units/mL) for different time-intervals (30 min, 1 day, 2 day, and 3 day). The GOx activity was probed through the oxidation of amplex red to fluorescent product resorufin as compared to the diffusional enzyme mixture in buffer solution.

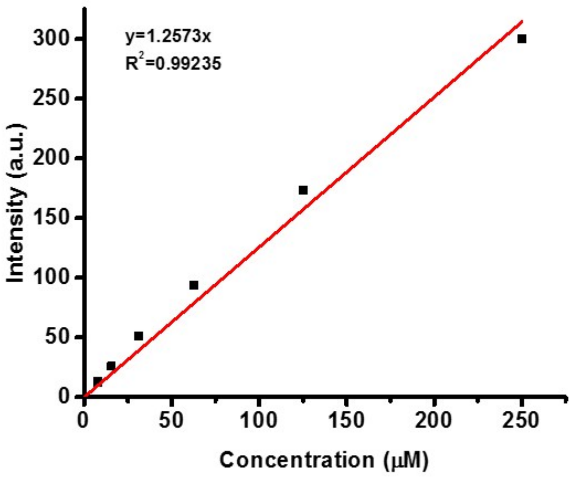

Figure S10. Calibration curve of the fluorescence intensity of coumarin in different concentration.

References

[1] N. G. Hentz, J. M. Richardson, J. R. Sportsman, J. Daijo, G. S. Sittampalam, *Anal. Chem*. **1997**, *69*, 4994-5000
